# *Kif14* Mutation Causes Severe Brain Malformation and Hypomyelination

**DOI:** 10.1371/journal.pone.0053490

**Published:** 2013-01-04

**Authors:** Kohei Fujikura, Tomiyoshi Setsu, Kenji Tanigaki, Takaya Abe, Hiroshi Kiyonari, Toshio Terashima, Toshiaki Sakisaka

**Affiliations:** 1 Division of Membrane Dynamics, Department of Physiology and Cell Biology, Kobe University Graduate School of Medicine, Kobe, Hyogo, Japan; 2 Division of Developmental Neurobiology, Department of Physiology and Cell Biology, Kobe University Graduate School of Medicine, Kobe, Hyogo, Japan; 3 Research Institute, Shiga Medical Center, Moriyama, Shiga, Japan; 4 Laboratory for Animal Resources and Genetic Engineering, RIKEN Center for Developmental Biology, Kobe, Hyogo, Japan; St. Jude Children's Research Hospital, United States of America

## Abstract

We describe a novel spontaneous mouse mutant, *laggard* (*lag*), characterized by a flat head, motor impairment and growth retardation. The mutation is inherited as an autosomal recessive trait, and *lag*/*lag* mice suffer from cerebellar ataxia and die before weaning. *lag*/*lag* mice exhibit a dramatic reduction in brain size and slender optic nerves. By positional cloning, we identify a splice site mutation in *Kif14*. Transgenic complementation with wild-type *Kif14*-cDNA alleviates ataxic phenotype in *lag/lag* mice. To further confirm that the causative gene is *Kif14*, we generate *Kif14* knockout mice and find that all of the phenotypes of *Kif14* knockout mice are similar to those of *lag/lag* mice. The main morphological abnormality of *lag/lag* mouse is severe hypomyelination in central nervous system. The *lag/lag* mice express an array of myelin-related genes at significantly reduced levels. The disrupted cytoarchitecture of the cerebellar and cerebral cortices appears to result from apoptotic cell death. Thus, we conclude that *Kif14* is essential for the generation and maturation of late-developing structures such as the myelin sheath, cerebellar and cerebral cortices. So far, no *Kif14*-deficient mice or mutation in *Kif14* has ever been reported and we firstly define the biological function of *Kif14 in vivo*. The discovery of mammalian models, *laggard*, has opened up horizons for researchers to add more knowledge regarding the etiology and pathology of brain malformation.

## Introduction

Mammalian brain development depends on a complex and highly regulated sequence of events, and can be divided into four overlapping processes: cell birth (neurogenesis), migration, formation of connections (including elaboration of processes, synapse formation, cell death and axonal regression) and myelination. Genetic mutations that affect the ability of neural cells to undergo these orderly and precisely paced developmental steps result in developmental arrest, often leading to death of the affected cell populations. Genetic mapping of such mutations in mice has led to the identification of proteins essential for neuronal migration, differentiation and survival. These mouse neurological mutants exhibit defects in a variety of processes, and include *open brain* (affecting neural tube development) [1], [2], *staggerer* and *lurcher* (causing degeneration of Purkinje cells and granule cells) [3], *weaver* and *reeler* (affecting neuronal migration) [4], [5], [6], and *jimpy* and *quaking* (affecting myelination) [7]. Similar developmental defects likely underlie human neurological disorders as well.

The myelin sheath allows for the rapid conduction of action potentials along the nerve fiber. In addition to facilitating saltatory conduction, the myelin sheath also insulates axons and contributes to their survival. Oligodendrocytes are the myelinating glial cells of the central nervous system (CNS), and are generated from oligodendrocyte precursor cells (OPCs), which are derived from progenitors in germinal areas of the CNS [8]. During CNS development, these precursors proliferate and migrate into the subcortical white matter, where they extend complex branched processes, and then initiate myelination [9]. *In vitro* studies demonstrate that oligodendrocyte development comprises several stages [10]. These stages are characterized by distinct morphological changes, unique cell surface antigen expression and differential responses to growth factors [11], [12]. Although several factors regulate the differentiation of OPCs into mature oligodendrocytes, little is known about the effectors that control the conversion of pre-myelinating oligodendrocytes into myelinating oligodendrocytes.

In this report, we describe a novel mouse mutation, *laggard* (*lag*), that appears to be brain specific and resembles human microcephaly. Mice homozygous for the *lag* mutation exhibit a reduction in brain size, severe hypomyelination, and dysgenesis of the cerebral and cerebellar cortices and the hippocampus. Mutant mice suffer from severe ataxia and tremors, and die within 3 week of birth. The reduced brain size in *lag* mutant mouse appears to result from a burst of apoptotic cell death that occurs during late neurogenesis. Through positional cloning, transgene rescue and gene targeting, the causative mutation is localized to the *Kif14* gene, encoding a kinesin motor family protein. This is the first report of *Kif14* function *in vivo* and no mutation in *Kif14* gene has ever been reported. Since these phenotypes are highly reminiscent of phenotypes observed in the human microcephaly, this mutant represents a valuable tool to further our studying of the human brain malformation and the molecular mechanisms underlying various neural disorders.

## Materials and Methods

### Animals

The spontaneous mutation *laggard* was discovered during generation of RA-RhoGAP deficient mice. 129/SVJ-derived RW4 embryonic stem (ES) cells were used according to the conventional gene targeting procedures. The germline chimeras were mated with BDF1 (C57BL/6N×DBA/2N F1) females, and resulting F1 mice were intercrossed to produce F2 generations. The *laggard* progenitor mouse was initially identified at this generation by a phenotype characterized by cerebellar ataxia. Then male F2 heterozygous mice were backcrossed to the C57BL/6J female six times to obtain mutant mice heterozygous for recessive mutation in a C57BL/6J background. BDF1 and C57BL/6J mice were purchased from CLEA Japan and Japan SLC, respectively.

pCAGGS-Kif14 transgenic mice (Acc. No. CDB0476T: http://www.cdb.riken.jp/arg/TG%20mutant%20mice%20list.html) was established as follows: To generate the Tg construct, the full-length Kif14 cDNA was subcloned into the pCAGGS vector. The Tg founder mice were generated by microinjenction of linearized constructs into fertilized eggs. For transgenic genotyping, the specific primers were designed within the CMV promoter of the Kif14 construct: CMV-Forward, 5′-CAACGACCCCCGCCCATTGAC-3′; Reverse, 5′-GAACGTGGGGCTCACCTCGAC-3′.

Kif14 knockout mice (Acc. No. CDB1079K: http://www.cdb.riken.jp/arg/mutant%20mice%20list.html) was established as follows: To disrupt Kif14 in ES cells, gene targeting was used to replace exon 5 with a Pr-neomycin resistance cassette. The targeting vector was electroporated into HK3i ES cells derived from the B6 strain [13]. The knockout mice were maintained on a B6 genetic background. To distinguish the wild-type allele from mutant allele, two separate sets of primers were designed for each allele. The oligonucleotide primers designed for the wild-type and the targeted alleles were as follows: WT-Forward, 5′- CTCGCACATCTCGTGAGAAC-3′; KO-Forward, 5′- GGTCCCTCGAAGAGGTTCAC-3′; common Reverse, 5′-CGTGAGTTGTAAAGACTCTAAGGTGA-3′. The size of PCR product from the wild-type allele is 494 bp, and the size of PCR product from the targeted allele is 279 bp. All experiments were carried out with the approval of the Committee on Animal Care and Welfare, Kobe University Graduate School of Medicine. All efforts were made to minimize the suffering animals. Mice were housed in a 12-hour light-dark cycle, and given free access to food and water. We monitored the mice carefully from the birth. Once the mice could not reach the food by themselves, we provided food and water on the floor of the cage. For the morphological and molecular analyses, the mice were sacrificed at P12–14, which was the symptomatic age showing marked reduction in motor performance. The mice used for the behavioral and survival analyses were sacrificed at the end-stage when a mouse was not able to right itself within 30 s when placed on one side. Mice were anesthetized via intraperitoneal injection with sodium pentobarbital (5 mg/100 g body weight).

### Stationary Beam Test

P12 littermate mice were placed on a 5-cm-wide platform and allowed to either explore or step down from the platform. The platform was placed on the floor. The duration that each mouse remained on the platform and the numbers of animals of each genotype were statistically analyzed. Eight independent sets of tests were performed on each mouse during one session [14]. The error bars were calculated from SD (standard deviation) for each experiment.

### Linkage Mapping

The *laggard* mouse arose spontaneously on the B6/D2/129 inbred strain background. The *laggard* mice were outcrossed to C57BL/6J strains to generate F1 progeny, and the resulting F1 mice were intercrossed to produce the F2 generation. At 2 weeks of age, F2 mice were scored for weight, brain size and behavior and sorted by phenotype. 31 and 58 F2 affected mice were used for rough and fine linkage analysis, respectively. Their tail DNA was extracted using the DNeasy Kit (QIAGEN GmbH, Hilden, Germany) and was PCR amplified using primers for 74 polymorphic microsatellite markers. The resultant PCR products were pooled and separated by capillary electrophoresis on the ABI 3130xl Genetic Analyzer (Applied Biosystems, Foster City, CA) as described [15], and the data were analyzed using LOD (Logarithm of Odds) score method. The parametric LOD score was calculated on the basis of the estimated number of mice with genotypes concordant with either the normal or mutant phenotype, using the formula LOD = log_10_[p(linkage)/p(nonlinkage)] [16].

### Next Generation Sequencing

Liver DNA was extracted from a *lag*/*lag* mouse using the DNeasy Kit. RNA baits were designed and used for SeqCap Target Enrichment (Nimblegen, Madison, USA) to capture sequence from a 133.659 to 141.025 Mb region on mouse chromosome 1. Target enrichment was followed by DNA amplification and the resulting fragments were sequenced by next generation sequencing using an Illumina GA IIa sequencer (Illumina, San Diego, USA). Tablet software was used to identify SNPs and indels between B6 and the *lag*/*lag* sequence.

### Detection of Splice Site Mutation in *Kif14*

PCR amplification of exon5 splice junction of *Kif14* was performed using the KOD FX Neo reagent (TOYOBO, Japan) on the Gene Amp PCR system 9700 (Applied Biosystems, Foster City, CA). Primers used for amplification were as follows: Kif14 mut Forward 5′-GTCAGTAAGCCCCAGAGAGC-3′ and Reverse 5′-GGGAGAGAAGCGTGAGGATA-3′. The amplified PCR products (*Mus musculus* strain C57BL/6J chromosome 1, GRCm38, 136,478,139–136,478,508) were purified and sequenced using the Big Dye Terminator V3.1 kit (Applied Biosystems) on an ABI Prism 3130xl Genetic Analyzer.

### Northern Blotting

100 ng of polyA RNA from P12 mouse whole brain (+/+, *lag*/+, *lag*/*lag*) were size-fractionated on a 0.8% denaturing formaldehyde agarose gel and transferred onto a positively-charged nylon membrane (Roche, Mannheim, Germany) by capillary transfer. Digoxigenin-labeled DNA probes for *Kif14* were generated with DIG-High Prime DNA labeling kit (Roche, Mannheim, Germany) according to the manufactures instructions. Primers used for amplification of specific probes were as follows: Kif14 Northern Forward 5′-ACACACTTCGCATAGCAGACA-3′ and Reverse 5′-GGTGACATTCATCAAAAGACCA-3′. The blots were hybridized overnight at 68°C in DIG EasyHyb solution (Roche) with high sensitivity strippable cDNA probes labeled with digoxigenin. After hybridization, membranes were washed, incubated with anti-DIG antibody, and treated with CDP STAR detection reagents (Roche). Finally the blots were imaged on a LAS 4000 (Fuji FILM, Japan).

### RT-PCR

*First-stranded cDNA was synthesized using SuperScript III cDNA synthesis kit (Invitrogen).* RT-PCR of Kif14 (exons 4–7) mRNA was performed using the KOD FX Neo reagent (TOYOBO, Japan) on the Gene Amp PCR system 9700 (Applied Biosystems, Foster City, CA). PCR reactions were carried out in a total volume of 15 µl containing 1 µl of first strand cDNA, and 1 µl of 10 µM forward and reverse PCR primers. Primers used for amplification of specific genes were as follows: Kif14 exons 4–7 Forward 5′- CAGCTACCATCTTGAAATGAGC-3′ and Reverse 5′- GCCGTGGCTCTCTGTTTATTT-3′. Thermocycling conditions were as follows: 2 min at 95°C, followed by 35 cycles of 95°C for 30 s, 58°C for 30 s and 68°C for 1 min.

### Real Time PCR

First-stranded cDNA was synthesized using SuperScript III cDNA synthesis kit (Invitrogen). Simultaneous real-time PCR reactions of 4 selected genes (*Kif14*, *Olig2*, *Pdgfra* and *Mbp*) were performed by using SYBR premix ExTaqII (Takara) in an ABI PRISM 7500 Sequence Detection System (Applied Biosystems). The mRNA detection procedure consisted of a denaturing step at 95°C for 1 min followed by 40 cycles of 30 s at 95°C and 34 s at 60°C. Four replicates were run for each gene and each sample. Relative changes in mRNA expression were calculated by the ΔΔC_t_ method, using the Sequence Detection System 2.06 software (Applied Biosystems). Expression levels of target genes were normalized to expression of β-actin since they were found to be more relatively constant in different control samples. Forward and reverse primers used in RT-PCR analyses were as follows: *Kif14* Forward 5′- TATGAGATCAGGACACTTAGTGGTG-3′ and Reverse 5′- CCCACGGTGTGAGTTTCTTC-3′; olig2 Forward 5′-CATCCCGGGGACAAACTG-3′ and Reverse 5′-GTAGAGGCGCCGAGTGTG-3′; pdgfra Forward 5′-CTGAAGAAGACGACCCCAAC-3′ and Reverse 5′-TTGAACGTCCTCCCTTTGAC-3′; mbp Forward 5′-ATCCAAGTACCTGGCCACAG-3′ and Reverse 5′-TCCCTTGTGAGCCGATTTAT-3′; β-actin Forward 5′-TTCTTTGCAGCTCCTTCGTTGCCG-3′ and Reverse 5′-TGGATGGCTACGTACATGGCTGGG-3′.

### Antibodies

The GST-fusion fragment of *Mus musculus* Kif14 (aa 137–390) was produced in *Escherichia coli*, purified, and used as an antigen to raise a polyclonal antibody in rabbit. The rabbit anti-Kif14 polyclonal antibody was affinity purified by using non-tagged Kif14 (aa 137–390) immobilized on Amino-link agarose beads (Pierce). MBP (Nichirei or Chemicon), MAG (Invitrogen), CNPase (Covance), β-tubulin (Sigma), α-tubulin (Sigma), Cleaved Caspase-3 (Cell Signaling), active Caspase-3 (Promega), BrdU (BD Pharmingen), Calbindin (Acris Antibodies GmbH), Cux1 (CDP) (Santa Cruz), Olig2 (IBL), PDGFRα (Cell Signaling) and Foxp2 (Abcam) antibodies were purchased from the indicated suppliers.

### Histological Analysis on Paraffin Embedded Sections

The mice were deeply anesthetized with sodium pentobarbital (5 mg/100 g body weight). They were sacrificed by a transcardial perfusion of 0.1 M phosphate buffer (PB) containing 0.9% NaCl (phosphate-buffered saline, PBS) for 5 min at room temperature, followed by 4% paraformaldehyde in PB for 15 min at 4°C. The brains were removed from the skull, and then post-fixed in 4% paraformaldehyde for 2 h at 4°C. After the brains were dehydrated and paraffin embedded, the sagittal and coronal paraffin sections of 4-µm thickness were prepared on a sliding microtome SM 2000R (LEICA) and mounted on a gelatin-coated glassslide. The sections were stained with hematoxylin and eosin or 0.1% cresyl violet (EM Science), dehydrated with a series of ethanol solution, and then coverslipped with HSR solution (Sysmex).

### Immunohistochemistry

The mice were anesthetized, fixed by transcardial perfusion of 4% paraformaldhyde in 0.1 M PB. The brains and the spinal cords were removed from the skull and the vertebral canal, and then immersed in the same fixative for additional 2 h. They were cryoprotected in 20% sucrose PB overnight. The frozen sections were cut coronally or sagittally on a freezing microtome (Yamato Koki) at 40-µm-thick. The sections were washed in 0.1 M PBS and immersed in blocking buffer (0.3% Triton in PBS (PBST) containing 1% BSA and 0.05% azide) for 1 h. They were then incubated in anti-Cux1 (rabbit polyclonal, 1∶4000 dilution), anti-Foxp2 (rabbit polyclonal, 1∶4000 dilution), anti-calbindin (mouse monoclonal, 1∶5000 dilution), and anti-cleaved caspase-3 (rabbit monoclonal, 1∶400 dilution) for 1 h at room temperature, and then overnight at 4°C. For bright field observation, the sections after incubation of the primary antibodies were incubated in biotinylated anti-rabbit IgG (1∶500 dilution) or biotinylated anti-mouse IgG (1∶500 dilution), and then in the VECTASTAIN ABC Reagent (Vector). They were reacted in diaminobenzidine (DAB). After washing in 0.1 M PBS, sections were mounted on glass slides pretreated with gelatin. The sections were coverslipped with HSR solution.

For fluorescence imaging, mice were perfused and sectioned, as described above. Immunohistochemical staining was performed with primary antibodies for 48 hours at 4°C after blocking for 1 h at room temperature with 4% skim milk (Becton Dickinson). Then the sections were incubated for 1 h at room temperature with secondary antibodies (1∶1000 dilution; Molecular Probes). The primary antibodies used were anti-Olig2 (1∶100 dilution) and anti-PDGFRα (1∶15 dilution) antibodies. Slides were examined with an Olympus confocal laser scanning microscope (FV-300, Olympus). Nissl staining was performed using NeuroTrace Fluorescent Nissl Stains (1∶100 dilution; Molecular Probes) according to the manufacturer's instructions.

### Electron Microscopy

Mice (n = 4) were perfused with 1% paraformaldehyde (PFA) and 1.25% glutaraldehyde in 0.1 M phosphate buffer (PB), pH 7.3. Brains and spinal cords were removed from the skull and vertebral canal. The cerebrum and cerebellum were coronally or sagittally cut into 1-mm-thick tissue slices with a razor blade. The samples were then postfixed in 1% PFA and 1.25% glutaraldehyde in 0.1 M PB for 1 h and then in 2% osmium tetroxide in PB for 1 h. Thereafter, the samples were washed with distilled water, stained overnight with 0.5% aqueous uranyl acetate at RT, dehydrated with ethanol, and embedded in Polybed 812 (Polyscience). Ultrathin sections were cut with a diamond knife, double stained with uranyl acetate and lead citrate, and observed at 100 kV accelerating voltage using a transmission electron microscope (JEM-1010; JEOL). Images were processed with Photoshop software.

### TUNEL Assay

Terminal deoxynucleotidyl transferase dUTP nick end-labeling (TUNEL) assays were performed using the DeadEnd Colorimetric TUNEL System (Promega, Madison, WI, USA) according to the manufacturer's instructions.

### BrdU Labeling Analysis

We injected pregnant mice intraperitoneally with BrdU (100 mg/kg) at E12.5 and E15.5, 2 h before sacrifice. The embryos were fixed in 4% PFA for 12 h and were dehydrated, embedded in paraffin. After the embryos were serially sectioned at 6 µm-thickness, a series of every 5 sections were pretreated in 2 N HCl for 1 h at 37°C, rinsed 3 times in PBS. They were then incubated with anti-BrdU (BD Pharmingen; 1∶500) for 24 h at 4°C, and 1 h with the biotinylated anti-mouse IgG (Vector; 1∶500), and the ABC-Kit for 30 min. Proliferating BrdU-positive neurons were visualized by using nickel-cobalt-DAB (0.05% 3,3' DAB, 0.025% nickel chloride, 0.025% cobalt acetate) resulting in dark blue staining.

### Cell Counts and Statistical Measures

BrdU, TUNEL, Cux1 and Foxp2-positive cells were counted in primary somatosensory cortex by T. S. to ensure that any observer error was consistent. Cell counts were performed by using serial coronal sections through the primary somatosensory cortex. The outline of the dorsal cortex and its subnulear structures were drawn with the aid of camera lucida attached to an Olympus microscope. Every forth section was examined with a 40× objective lens and a net ocular micrometer (10×10 grid measuring 1×1 mm in width) in a 10× ocular lens. Nuclei of the positive neurons were plotted on a tracing of the primary somatosensory cortex. Olig2 and PDGFRα-positive cells were counted in primary somatosensory cortex by K. T. Every section was examined with a net ocular micrometer (10×10 grid measuring 1×1 mm in width). Oligodendrocytes in the cross section of optic nerve were counted by using 1 µm-thickness semithin section of the optic nerve. Identification of oligodendrocytes in the semithin section was confirmed by transmission EM observation of the adjoining ultrathin section. The error bars were calculated from SD for each experiment and the statistical significance was calculated using the program Student’s *t*-test.

### Microarray Analysis

Total RNA was extracted from P14 mouse cerebrum using TRIzol (Invitrogen, Carlsbad, CA) and followed by a cleanup step using PureLink RNA Mini Kit (Invitrogen, Carlsbad, CA). The RNA was processed at the Takara Bio Genomics Center for hybridization with Affymetrix GeneChip Mouse Genome 430 2.0 Array. Raw data from the microarrays were calculated using Microarray Suite version 5.0 (MAS5.0) with Affymetrix default setting and global scaling as the normalization method. The data files for microarray are available from GEO under accession number GSE38573.

### Other Procedures

Protein concentrations were determined with bovine serum albumin as a reference protein [17]. SDS-PAGE was done as described [18].

## Results

### General Appearance of *laggard* Mice

**Figure 1:**
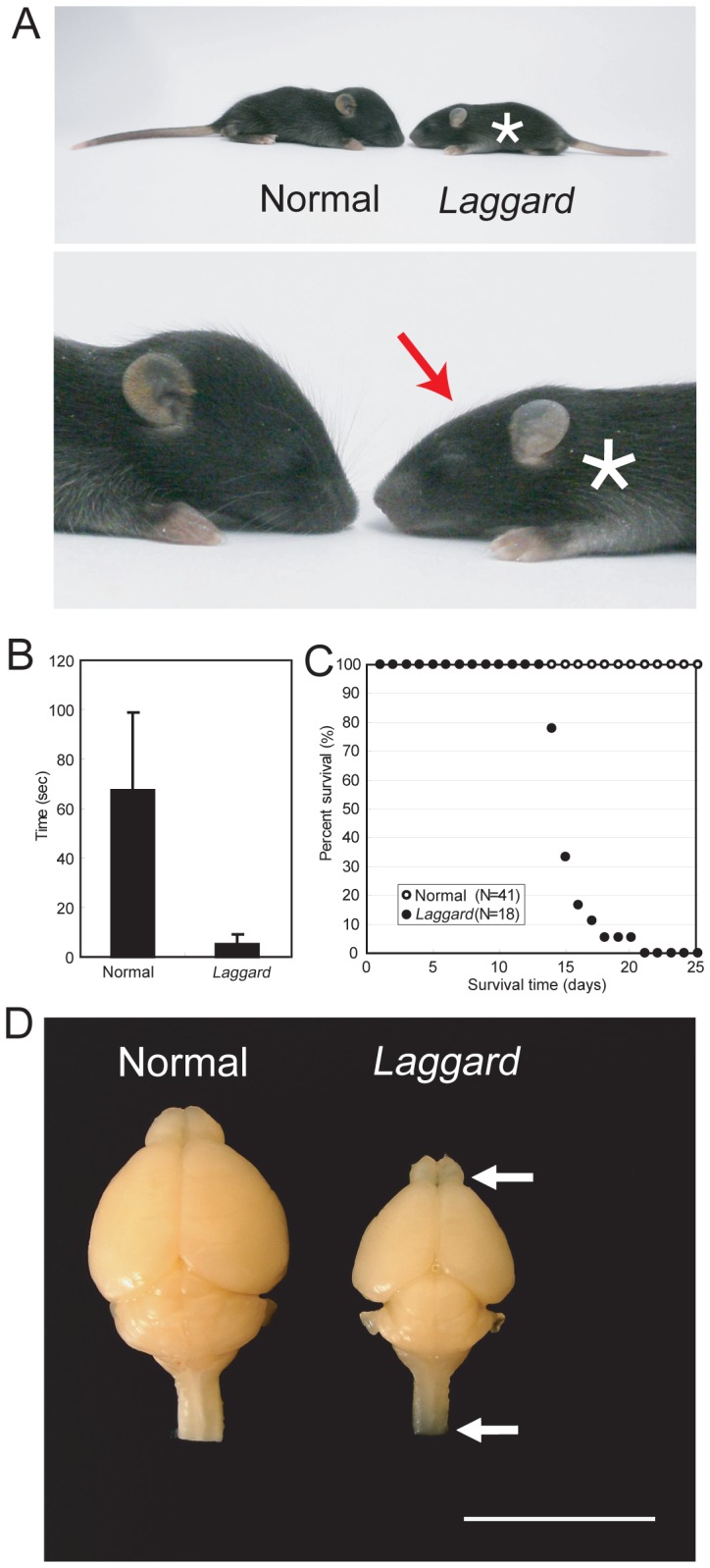
*lag* mutation preferentially affects the brain. (A) The *lag* mutant phenotype. The littermate normal control mouse and the *lag* mutant mouse faced each other. Upper panel: general appearance. Lower panel: enlarged head image. Asterisk indicates the mutant mouse and red arrow indicates the flat head of the mutant mouse. (B) Behavior test for ataxia. Quantitative analysis of the duration the littermate normal control mice (n = 16) and the *lag* mutant mice (n = 16) stood on a narrow platform. Error bars represent SD. (C) Survival curves. Survival rate of the littermate normal control mice (open circles, n = 41) and the *lag* mutant mice (closed circles, n = 18) from postnatal day 1 (P1) to P25. (D) Whole brain images of the littermate normal control mouse and the *lag* mutant mouse at P12. Arrowheads indicate the translucent olfactory bulb and spinal cord of the *lag* mutant brain. Bar, 10 mm.

During the generation of some knockout mice, we obtained a line in which mice exhibited small head, tremor, ataxic gait and uncoordinated locomotion during the early postnatal week (Figure 1A and Video S1). This mutation, which we designated *laggard* (*lag*), was transmitted in an autosomal recessive manner. We initially assumed that *lag* was caused by gene disruption due to misinsertion of the targeting vector. However, *lag* was independent of gene targeting vector integration, suggesting that the mutation arose spontaneously. *lag/lag* mice appeared in crosses at the expected Mendelian frequency. At birth, *lag/lag* mice were indistinguishable from normal littermates (+/+ and *lag*/+ mice). However, by postnatal day 10 (P10), the mutants exhibited an overt ataxic phenotype that increased in severity over time. At P12, *lag/lag* mice were unable to stand for 10 s on a narrow (5-cm-wide) platform (stationary beam test [14]), whereas normal littermates maintained their balance for 1 min (Figure 1B). Their gait was wide and uncoordinated, and they frequently fell on their backs while walking. These defects in voluntary movement control, posture and balance were accompanied by progressive weakness and failure to gain body weight. By P14, the homozygous mutants in a litter could be identified by their small size and uncontrolled movements. The homozygous mutants all died by P21 (Figure 1C).

We compared body and brain weights of *lag/lag* mice with those of unaffected littermates (+/+ and *lag*/+ mice) at various ages. Although *lag/lag* mice often weighed slightly less than their unaffected littermates in the first week of life, their body weights fell within the range of normal variation during this period (Figure S1A). However, after the first week, differences in body size increased until the death of the animals. Strikingly, brains were much smaller in *lag/lag* mice compared with their unaffected littermates (Figure S1B). At P1, *lag/lag* brains weighed 25% less than those of their unaffected littermates, and by P9, the difference in brain size increased to 47%. After this time point, the *lag/lag* brain remained approximately one-half the size of a normal brain throughout the 2–3 week life-span. Numerous regions were affected, contributing to the overall decrease in brain size. Most dramatic was the reduction in the size of cerebral and cerebellar cortices and olfactory bulb (Figure 1D). The superior and inferior colliculi were exposed due to the cerebellar hypoplasia. The parenchyma of the mutant olfactory bulb and that of the spinal cord appeared transparent. The gross morphologies of other organs were indistinguishable between *lag/lag* and unaffected animals (data not shown).

### *laggard* is a Novel Allele of the Kif14 Gene

**Figure 2:**
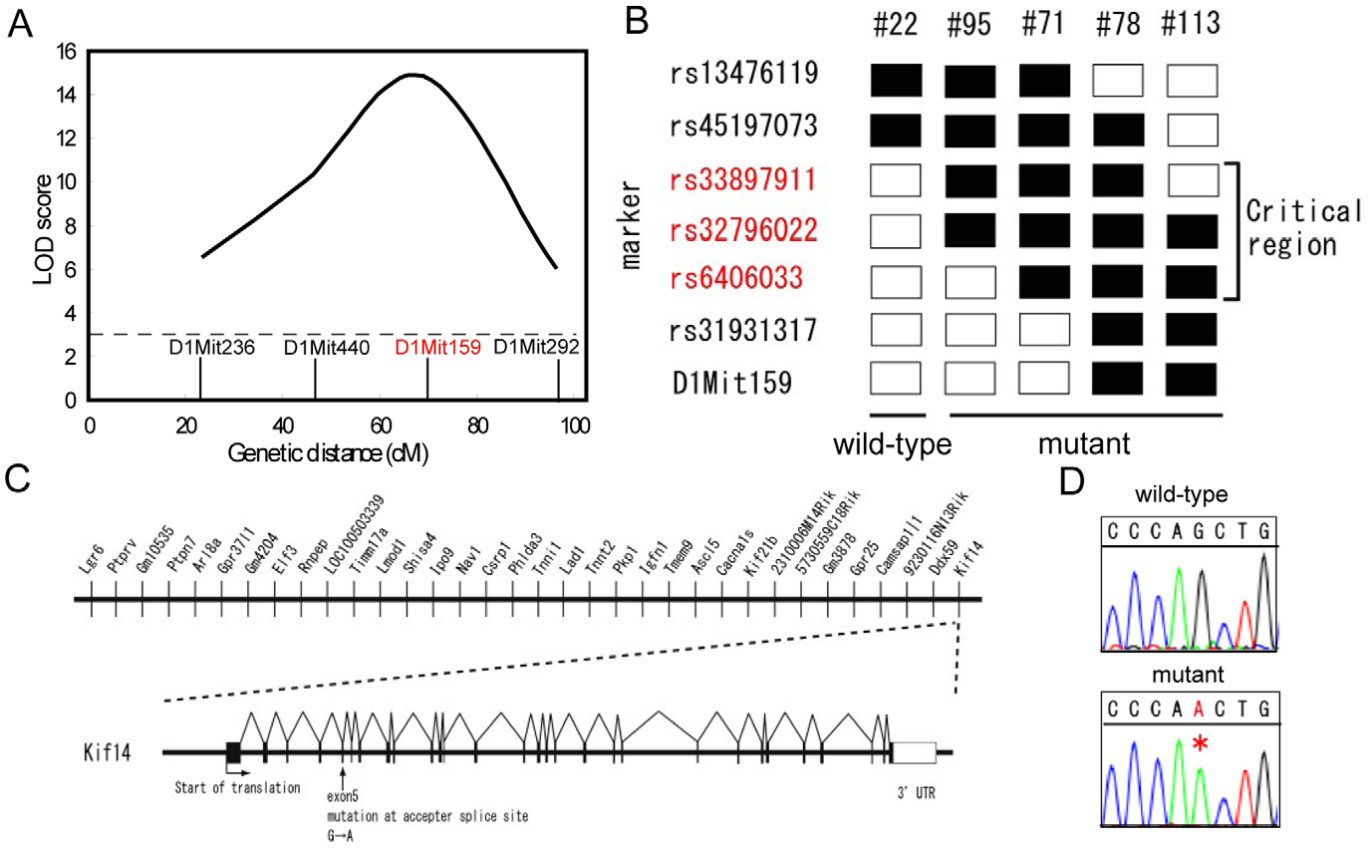
Positional cloning of *Kif14* in the *lag* mutant mouse. (A) LOD score plot of the *lag* locus on chromosome 1 for the F2 intercrosses. The microsatellite markers are shown on the lower side. (B) Fine mapping analysis of the *lag* locus. Black boxes represent DBA/2N allele homozygotes; white boxes represent C57BL/6 allele homozygotes and heterozygotes. The identification number of each mouse is listed at the top (# number). (C) Candidate genes mapped to the crucial region. *Kif14* exon-intron structure, positions of the 3′ UTR, and start site of translation are indicated according to the NCBI reference assembly build 37. (D) G/A substitution at the 3′ splice acceptor site of *Kif14* exon 5. The genomic sequence analysis of exon5 splice junction of *Kif14* gene was performed. The red asterisk denotes the mutation site for *lag*.

To understand the molecular basis of the *laggard* phenotype, a linkage mapping approach was used to identify the causative mutation. Heterozygous carriers of the spontaneous mutation, which arose on the 129/SVJ, C57BL/6N, DBA/2N (2∶1∶1) background, were backcrossed to C57BL/6J mice to generate C57BL/6J background F1 heterozygous carriers. An intercross breeding strategy was used to produce F2 mutants, since homozygous mutants died by P21 and thus were unable to mate. Rough mapping using genome-wide sets of 74 polymorphic microsatellite markers was conducted for wild-type and mutant F2 mice. In order to identify the causative locus for *laggard*, the parametric LOD score was calculated on the basis of the number of mice with genotypes concordant with either the normal or mutant phenotype. A very strong association (peak LOD score = 14.82) was detected at D1Mit159 (Figure 2A), whereas on other chromosomes no evidence of linkage was found. In a second intercross with C57BL/6J mice, the genotyping of F2 mice for polymorphic microsatellite markers spaced throughout the genome confirmed the mutation's location on the distal end of chromosome 1. Fine mapping with additional markers further localized the mutation to the 1.5-Mb region between the SNP markers *rs33897911* and *rs6406033* (Figure 2B). This region included 34 known and predicted genes at the time of mapping (Figure 2C). Of these, 10 genes were excluded because the corresponding knockout mouse lines have been reported to survive to adulthood, and they do not appear to have cerebellar ataxia. We compared the sizes and transcript levels of the remaining 24 genes in the brains of wild-type and *lag/lag* mice at P14 using reverse transcription-polymerase chain reaction (RT-PCR) with primer sets amplifying the complementary DNA (cDNA) between the 5′ and 3′ untranslated regions (UTRs), and found that the transcripts were indistinguishable in expression level or size.

We then sequenced genomic DNA within the 1.5-Mb region using next generation sequencer, and a G→A substitution (DDBJ accession number: AB733452) was identified at the 3′ splice acceptor site of exon 5 of the *Kif14* gene, which appears to disrupt splicing (data not shown). To confirm the splice site mutation, the genomic sequence analysis of exon5 splice junction of *Kif14* gene was performed (Figure 2D). The mutation in *Kif14* gene arouse on a DBA/2N genetic background. To rule out the possibility that the G→A substitution represents natural variation among different strains, we sequenced the parental BDF1 (C57BL/6N×DBA/2N F1) strain and searched the mouse genome database. The G→A substitution was never detected (data not shown). Kif14 is a kinesin motor family protein, which is expressed in multiple tissues [19]. Recent reports using small-interfering RNA (siRNA)-mediated knockdown in cultured non-neuronal cells suggest that *Kif14* is involved in cytokinesis [20]. However, the role of *Kif14* in living animals is unclear.

**Figure 3:**
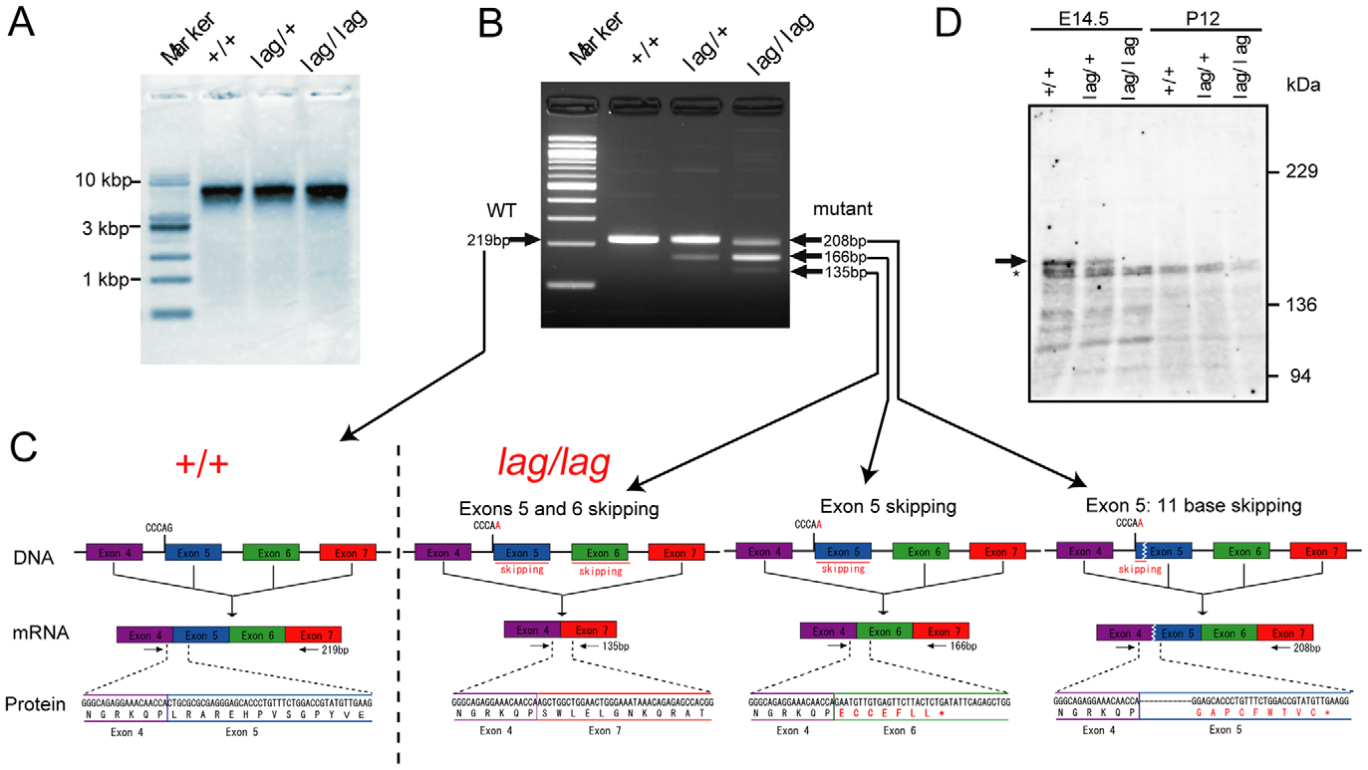
Identification of exon skipping in mRNA from *lag/lag* mouse. (A) Northern blot analysis of *Kif14* mRNA. PolyA RNA from whole brains of +/+, *lag*/+, *lag*/*lag* mice was subjected to agarose gel-electrophoresis and then transferred to a nylon membrane. The membrane was hybridized with a *Kif14* cDNA probe. (B) Transcripts analysis of *Kif14* splice acceptor site mutation. Agarose gel-electrophoresis of *Kif14* PCR products from first-strand cDNA prepared from whole brains of +/+, *lag*/+, *lag*/*lag* mice. (C) Sequence analysis of the three *Kif14* transcripts identified in the *lag*/*lag* mouse. The *Kif14* transcript in the *lag*/*lag* mouse exhibited skipping of an 11-bp segment of exon 5, entire exon 5, and exons 5 and 6 as a result of a G to A substitution at the acceptor site of exon 5. (D) Western blot analysis of Kif14 protein at E14.5 and P12. Whole brain extracts (40 µg of proteins) from +/+, *lag*/+, *lag*/*lag* mice at E14.5 and P12 were subjected to SDS-PAGE, followed by immunoblotting with the anti-Kif14 rabbit polyclonal antibody. Arrow indicates full-length Kif14. Asterisk indicates the non-specific band.

Northern blot analysis demonstrated that *Kif14* mRNA size were indistinguishable between +/+, *lag/*+ and *lag/lag* mice at P12 (Figure 3A). Quantitative real-time PCR showed that the expression level of *Kif14* mRNA was not noticeably different between *lag*/*lag* mice and littermate controls. We then compared the sizes of the transcripts in the brains of +/+, *lag/*+ and *lag/lag* mice at P12 using RT-PCR with primer sets amplifying the partial cDNA between exons 4 and 7. A single band, four bands, and three bands were amplified using mRNA from +/+, *lag*/+ and *lag*/*lag* mice, respectively (Figure 3B). We cloned and sequenced these bands, and found 3 products generated by exon skipping. The mutation at the splice acceptor site of exon 5 results in 3 Kif14*^lag^* transcripts; exon 5 skipping (DDBJ accession number: AB733449), exons 5 and 6 skipping (DDBJ accession number: AB733450), and 11-bp skipping (DDBJ accession number: AB733451) (Figure 3C and S2A). Exon 5 skipping and 11-bp skipping introduced premature stop codons. Exons 5 and 6 skipping did not cause a frameshift in the open reading frame, resulting in a truncated protein lacking the 28-aa region that forms the microtubule-binding domain. We raised a Kif14-specific polyclonal antibody and could detect wild-type Kif14 and three Kif14*^lag^* proteins by western blot analysis (Figure S2B). We confirmed by western blot analysis that the wild-type Kif14 protein cannot be detected in the *lag/lag* brain (Figure 3D). Kif14*^lag^* proteins were not detected in the *lag/lag* brain, but detected in the Kif14*^lag^*-overexpressing cultured cells (Figure S2 and S3), suggesting that endogenous Kif14*^lag^* proteins are much less stable in *lag/lag* brain than Kif14*^lag^* proteins overexpressed in cultured cells.

### Expression of Transgenic *Kif14* Alleviates Ataxic Phenotype in *lag/lag* Mice

**Figure 4:**
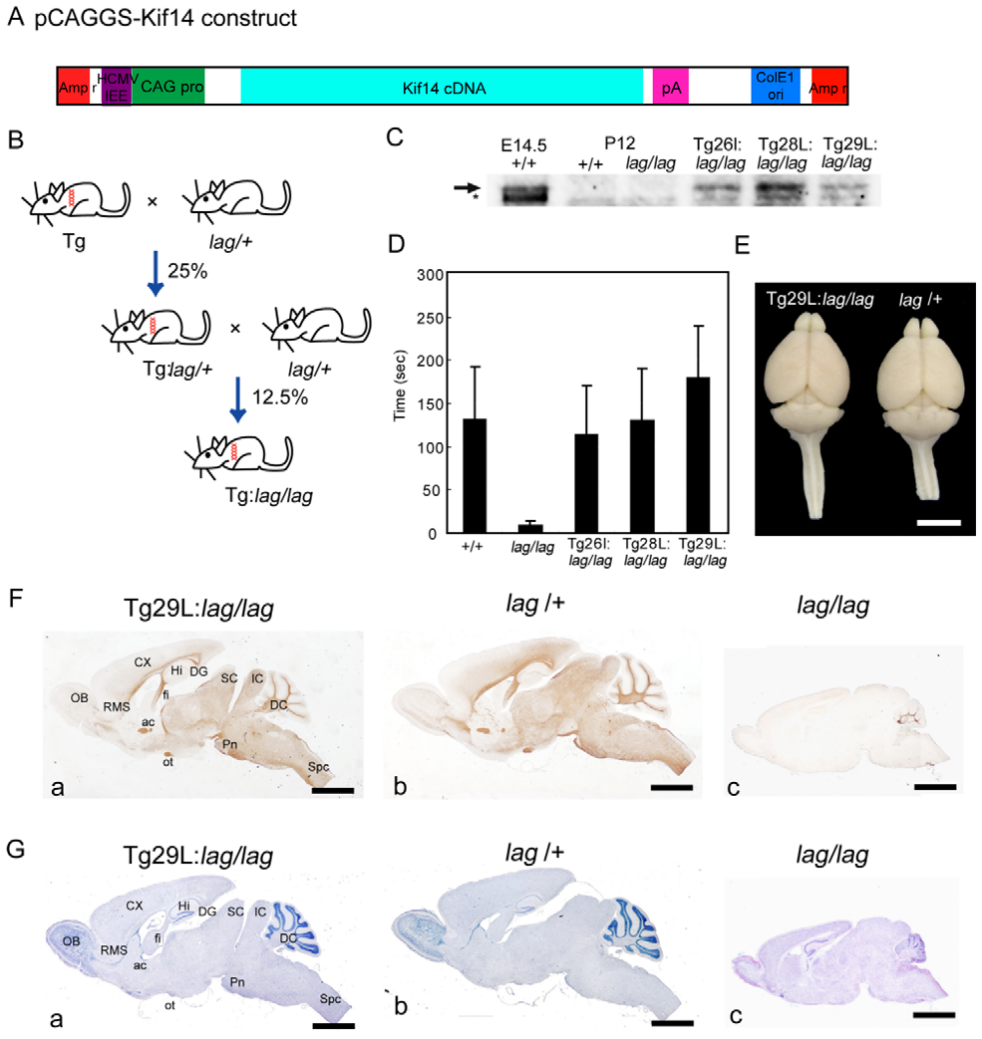
*Transgene rescue.* (A) The transgenic rescue construct. pCAGGS-Kif14 carries a strong constitutive promoter (CAG pro, CAG promoter), mouse *Kif14* cDNA, SV40 early splice region/polyadenylation signal (pA), human cytomegalovirus immediate early enhancer (HCMVIEE), ampicillin resistance gene (Amp r) and E. coli replicate origin (ColE1 ori). (B) Transgenic mice were crossed with *lag*/+ mice to yield Tg:*lag*/+ mice. Tg:*lag*/+ mice were crossed with *lag*/*lag* mice to obtain Tg mice in a *lag*/*lag* background. (C) Expression levels of Kif14 in three lines of Tg:*lag*/*lag* mice. Whole brain extracts (20µg of proteins) from E14.5 wild-type mouse, P12 wild-type mouse, P12 *lag*/*lag* mouse, and P12 Tg:*lag*/*lag* mice lines (Tg26l:*lag*/*lag*, Tg28L:*lag*/*lag*, and Tg29L:*lag*/*lag*) were subjected to SDS-PAGE, followed by immunoblotting with the anti-Kif14 rabbit polyclonal antibody. Arrow indicates full-length Kif14. Asterisk indicates the non-specific band. (D) Behavior test for ataxia. Quantitative analysis of the duration the littermate wild type (+/+) mice (n = 16), the *lag* mutant (*lag/lag*) mice (n = 16), and the Tg:*lag*/*lag* mice (Tg#26l:*lag*/*lag*, n = 16; Tg#28L:*lag*/*lag*, n = 16; Tg#29L:*lag*/*lag*, n = 16) stood on a narrow platform. Error bars represent SD. (E) Dorsal views of P16 whole-brain from *Kif14* transgenic rescue (Tg29L:*lag/lag*) and non-transgenic littermate normal control mouse (*lag/*+) mice. Bar, 5 mm. (F) Sagittal brain sections from *Kif14* transgenic rescue (Tg29L:*lag/lag*) mouse (Fa), non-transgenic littermate normal control mouse (*lag/*+) (Fb), and *lag* mutant mouse (*lag/lag*) (Fc), at P16 were immunostained with the anti-MBP antibody. Bar, 2 mm. (G) Sagittal brain sections from *Kif14* transgenic rescue (Tg29L:*lag/lag*) mouse (Ga), non-transgenic littermate normal control mouse (*lag/*+) (Gb), and *lag* mutant mouse (*lag/lag*) (Gc), at P16 were counterstained with hematoxylin. ac, anterior commissure; CX, cortex; DC, deep cerebellar nuclei; DG, dentate gyrus; fi, fimbria; Hi, hippocampus; Ic, inferior colliculus; OB, olfactory bulb; ot, optic tract; Pn, pontine nuclei; RMS, rostral migratory stream; Sc, superior colliculus; Spc, spinal cord. Bars, 2 mm.

To confirm that *Kif14* was the *lag* gene, we tested whether transgenic (Tg) expression of Kif14 could rescue the *lag* phenotype. We made a transgene in which a modified chicken β-actin promoter (CAG promoter) [21] drove the expression of the wild-type *Kif14* cDNA (DDBJ accession number: AB731728) (Figure 4A). The wild-type *Kif14* cDNA, under the regulation of the CAG promoter, was injected into fertilized eggs from BDF1 mice. We obtained 3 independent Tg lines (#26l, #28L and #29L) that expressed wild-type Kif14 protein. We then mated them with *lag*/*+* mice to obtain Tg mice in the *lag*/*lag* background (Figure 4B). We obtained 3 independent Tg lines (Tg#26l:*lag*/*lag*, Tg#28L:*lag*/*lag* and Tg#29L:*lag*/*lag*) in the *lag*/*lag* background. These transgenic lines expressed wild-type Kif14 protein at different levels (Figure 4C). Considerable improvement of the ataxic phenotype was observed in the Tg#26l:*lag*/*lag*, Tg#28L:*lag*/*lag* and Tg#29L:*lag*/*lag* mice. These mice could remain balanced for 2 min on a narrow (5-cm-wide) platform, whereas their littermate *lag*/*lag* mice were unable to remain standing for 10 s (Figure 4D). The size of cerebral and cerebellar cortices of *Kif14* transgenic rescue mouse (Tg29L:*lag*/*lag*) is apparently similar to that of non-transgenic littermate normal mouse (*lag*/+) (Figure 4E). The parenchyma of the *Kif14* transgenic rescue mouse (Tg29L:*lag*/*lag*) olfactory bulb and spinal cord are apparently similar to that of non-transgenic littermate normal mouse (*lag*/+). We also confirmed rescue of the *lag* phenotype (which we describe later) by immunostaining with the anti-MBP antibody and by Nissl staining (Figure 4F, G). These results strongly suggest that *Kif14* is the causative gene of the *lag* mutation.

### Generation and Characterization of *Kif14* Knockout Mice

**Figure 5:**
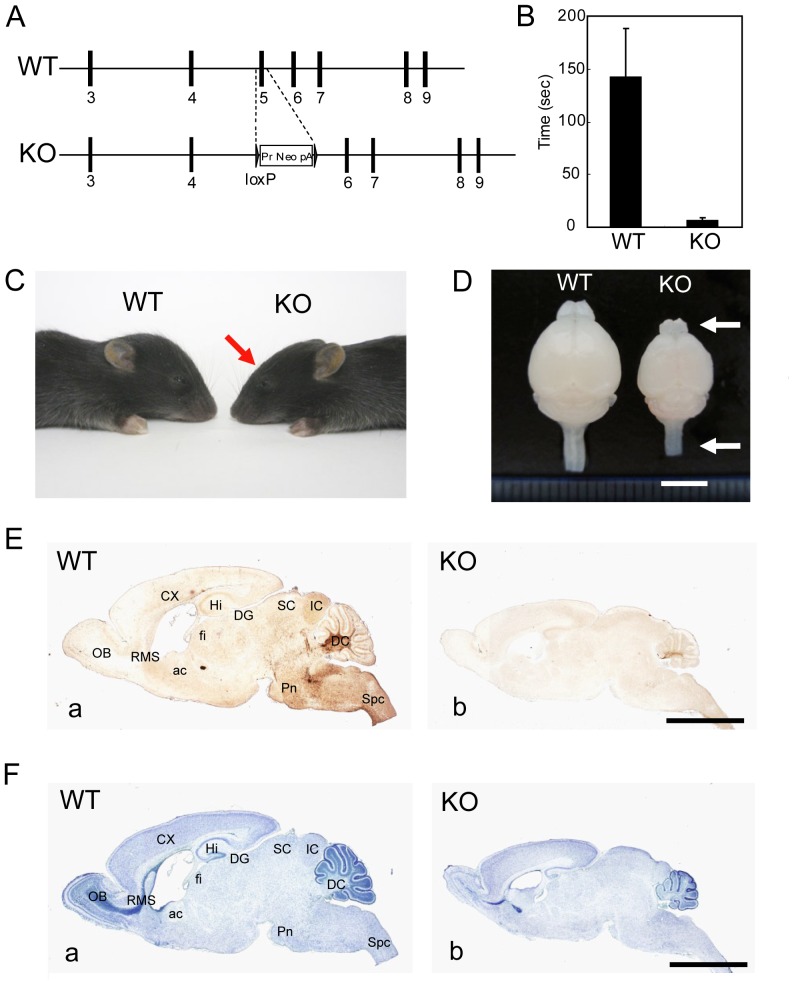
Gene targeting. (A) Schematic of the KO allele, in which exon 5 was deleted by homologous recombination in ES cells. (B) Behavior test for ataxia. Quantitative analysis of the duration the littermate wild-type mice (n = 8) and the *Kif14* KO mice (n = 8) stood on a narrow platform. Error bars represent SD. (C) The *Kif14* KO mouse phenotype. The littermate wild-type control mouse and the *Kif14* KO mouse faced each other. Red arrow indicates the flat head of the *Kif14* KO mouse. (D) Whole brain images of the littermate wild-type control mouse and the *Kif14* KO mouse at P12. Arrowheads indicate the translucent olfactory bulb and spinal cord of the *Kif14* KO brain. Bar, 5 mm. (E) Sagittal brain sections from the littermate wild-type control mouse (Ea) or *Kif14* KO mouse (Eb) at P12 were immunostained with the anti-MBP antibody. Bars, 2 mm. (F) Sagittal brain sections from the littermate wild-type control mouse (Fa) or *Kif14* KO mouse (Fb) at P12 were counterstained with hematoxylin. ac, anterior commissure; CX, cortex; DC, deep cerebellar nuclei; DG, dentate gyrus; fi, fimbria; Hi, hippocampus; Ic, inferior colliculus; OB, olfactory bulb; Pn, pontine nuclei; RMS, rostral migratory stream; Sc, superior colliculus; Spc, spinal cord. Bars, 2 mm.

To further confirm that the *lag* gene encodes *Kif14*, we deleted exon 5 from the allele by a gene-targeting technique in embryonic stem (ES) cells and generated mice homozygous for the targeted allele (Figure 5A). The homozygous mice appeared in crosses at the expected Mendelian frequency. At birth, homozygous mice were indistinguishable from wild-type littermates. By P10, the homozygous mice exhibited a cerebellar ataxic phenotype that increased in severity over subsequent days, similar to *lag*/*lag* mice. At P12, *Kif14* knockout mice were unable to stand for 10 s on a narrow (5-cm-wide) platform, whereas wild-type littermates stayed balanced for 2 min (Figure 5B). Their gait was wide and uncoordinated, and they frequently fell on their backs while walking. They also displayed a flathead, a reduction in brain size, slender optic nerves, and a translucent spinal cord, suggesting a lack of myelination (Figure 5C, D). We confirmed hypomyelination in the central nervous system and disrupted cytoarchitecture of the cerebellar and cerebral cortices and the hippocampus (which we describe later) by immunostaining with the anti-MBP antibody and by Nissl staining (Figure 5E, F). All of these phenotypes were similar to those of *lag*/*lag* mice. Thus, we conclude that the *lag* phenotype is caused by *Kif14* disruption.

### Hypomyelination in *lag/lag* Mice

**Figure 6:**
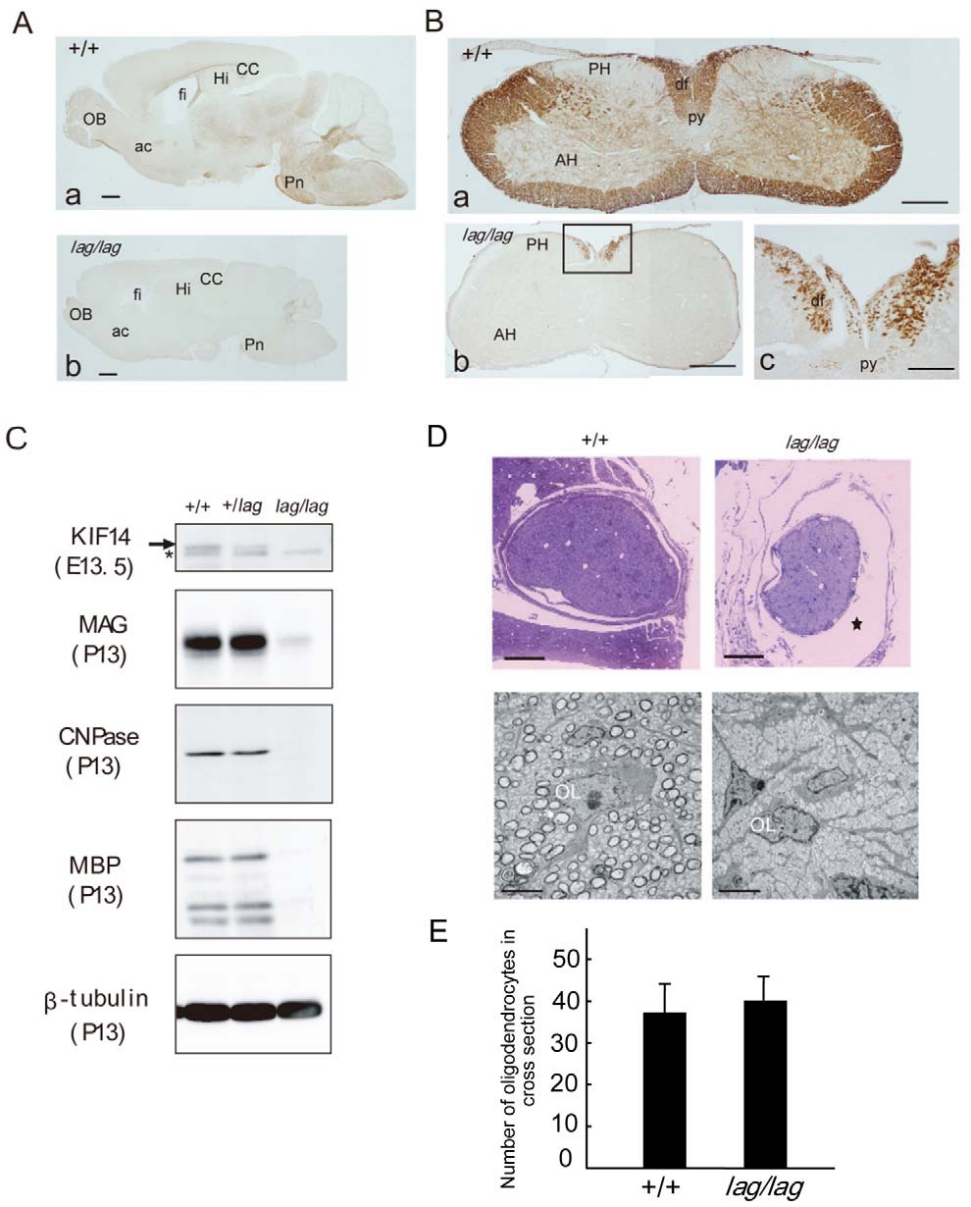
Hypomyelination in *lag/lag* mice. (A) Littermate wild type (Aa) and *lag/lag* mutant (Ab) whole brain sagittal sections at P14 were immunostained with the anti-MBP antibody. ac, anterior commissure; cc, corpus callosum; fi, fimbria; Hi, hippocampus; OB, olfactory bulb; Pn, pontine nuclei. Bars, 1 mm. (B) Littermate wild type (Ba) and *lag* mutant (Bb) spinal cord coronal sections at P14 were immunostained with the anti-MBP antibody. (Bc) is an enlarged image of the boxed area in (Bb). AH, anterior funiculus; df, dorsal funiculus; PH, posterior funiculus; py, pyramidal tract. Bars in (Ba) and (Bb), 1 mm. Bar in (Bc), 200 µm. (C) Protein levels of various myelin components in the brains of both the littermate wild type and the *lag* mutant mice. Myelin-related proteins from whole brain extracts from +/+, *lag*/+ and *lag*/*lag* mice were analyzed by quantitative immunoblotting with various antibodies against the indicated proteins. 18 µg of protein for Kif14 detection. 12 µg of protein for MAG, CNPase, MBP, and β-tubulin detection. Arrow indicates full-length Kif14. Asterisk indicates the non-specific band. (D) Oligodendrocyte morphology in the littermate wild type and the *lag* mutant mice. Upper panels: semi-thin sections of optic nerves were stained with toluidine blue. Star indicates enlargement of subarachnoid space. Bars, 1 mm. Lower panels: higher magnification of the optic nerves using transmission electron microscopy. OL, oligodendrocyte. Bars, 5 µm. (E) The number of oligodendrocytes in cross section of optic nerve (n = 6). Error bars represent SD.

The *lag*/*lag* mice displayed a translucent spinal cord, suggesting a lack of myelination (Figure 1D). To clarify the myelin deficit in these mutants, brain and spinal cord sections were immunostained with anti-MBP antibody. In sagittal sections of wild-type brain, MBP-immunoreactive nerve fibers were detected in the corpus callosum, fimbria hippocampi, anterior commissure, corpus medullare of the cerebellum, internal capsule, cerebral peduncle and pyramis (Figure 6A). However, in the mutant mouse brain, all of these fibers were MBP-negative, although weakly labeled fibers were scattered in the corpus medullare of the cerebellum. In transverse sections of the normal cervical spinal cord, ascending and descending fibers in the white matter were strongly immunostained with anti-MBP antibody (Figure 6B). However, in the mutant cervical cord, no MBP-immunolabeled fibers were found in the ventral or lateral funiculi. Although a few myelinated fibers were identified in the dorsal half of the dorsal funiculus, descending fibers in the pyramidal tract were not immunostained. Consistent with the immunohistochemical data, western blot analysis of total brain protein extracts demonstrated that the myelin proteins MBP, CNPase and MAG were dramatically reduced in the mutant brain (Figure 6C). We next examined the expression levels of *Kif14* in wild-type mouse brains at different developmental stages and found that the expression of *Kif14* was limited between E12.5 and E16.5 (Figure S4).

To identify genes whose expression levels were affected by the *lag* mutation, total RNA samples isolated from P14 wild-type and mutant cerebra were analyzed by microarray using the Affymetrix GeneChip Mouse Genome 430 2.0 Array. Table 1 shows the genes that displayed a greater than two-fold decrease in expression level in the mutant compared with the wild-type. This list predominantly contains genes known to play a role in myelination, including structural proteins and lipid-synthesizing enzymes. Strikingly, a comparison of this list with a database of genes expressed in the oligodendrocyte lineage [22] revealed that all of the genes in Table1 are up-regulated during oligodendrocyte maturation. These findings suggest that *lag* is likely to be involved, either directly or indirectly, in coordinating the myelination program in oligodendrocytes.

**Table 1:** Data of microarray analysis on RNA samples isolated from brains of P14 wild-type and mutant *laggard* mice.

Gene Title	Fold change
Myelin basic protein (Mbp)	−278.43
Myelin-associated oligodendrocytic basic protein (Mobp)	−239.47
Myelin oligodendrocyte glycoprotein (Mog)	−202.39
POU domain, class 4, transcription factor 2 (Pou4f2)	−145.62
Interferon activated gene 203 (Ifi203)	−138.13
Canopy 1 homolog (Cnpy1)	−112.55
Ectonucleotide pyrophosphatase/phosphodiesterase 6 (Enpp6)	−51.48
Cytochrome P450, family 3, subfamily a, polypeptide 41A (Cyp3a41a)	−50.89
Small nuclear ribonucleoprotein N (Snrpn)	−37.81
Proteolipid protein 1 (Plp1)	−36.79
Poly (ADP-ribose) polymerase family, member 1 (Parp1)	−36.22
Myb-like, SWIRM and MPN domains 1 (Mysm1)	−35.38
A disintegrin-like and metallopeptidase with thrombospondin type 1 motif, 4 (Adamts4)	−27.40
Breast carcinoma amplified sequence 1 (Bcas1)	−26.79
Leucine rich repeat containing 67 (Lrrc67)	−26.56
Guanylate binding protein 1 (Gbp1)	−25.53
Pyridoxal-dependent decarboxylase domain containing 1 (Pdxdc1)	−24.56
Paired-like homeobox 2b (Phox2b)	−24.53
Transmembrane protein 125 (Tmem125)	−24.42
Mitogen-activated protein kinase kinase kinase kinase 3 (Map4k3)	−18.48
G protein-coupled receptor 17 (Gpr17)	−18.40
G protein-coupled receptor 115 (Gpr115)	−18.25
UDP galactosyltransferase 8A (Ugt8A)	−16.01
Myelin-associated glycoprotein (Mag)	−14.33
SRY-box containing gene 10 (Sox10)	−7.22
Fatty acid 2-hydroxylase (Fa2h)	−6.37
Claudin 11 (Cldn11)	−5.81
2',3'-cyclic nucleotide 3' phosphodiesterase (Cnp)	−5.39
Ermin (Ermn)	−4.76
Myelin and lymphocyte protein (Mal)	−3.81
Gelsolin (Gsn)	−2.92
G protein-coupled receptor 149 (Gpr149)	−2.70
Galactose-3-O-sulfotransferase 1 (Gal3st1 )	−2.63
Elongation of long chain fatty acids (Elovl7)	−2.46
Quaking (Qk)	−2.41
Oligodendrocytic myelin paranodal and inner loop protein (Opalin)	−2.26
Myelination associated SUR4-like protein (Masr)	−2.15
Aspartoacylase (Aspa)	−2.13
Sphingomyelin synthase 1 (Sgms1)	−2.12
Oligodendrocyte transcription factor 2 (Olig2)	−1.86
Oligodendrocyte transcription factor 1 (Olig1)	−1.85

Examination of optic nerves by transmission electron microscopy revealed that the vast majority of P14 mutant axons were unmyelinated in sharp contrast with the wild-type (Figure 6D). The number of oligodendrocytes in cross section of the mutant mouse optic nerve (37.8±7.13) was similar to that in the wild-type mouse optic nerve (40.8±5.18) (Figure 6E). Although mutant cells extended normal-looking processes that were in contact with axons, the majority of these processes did not form myelin sheaths.

**Figure 7:**
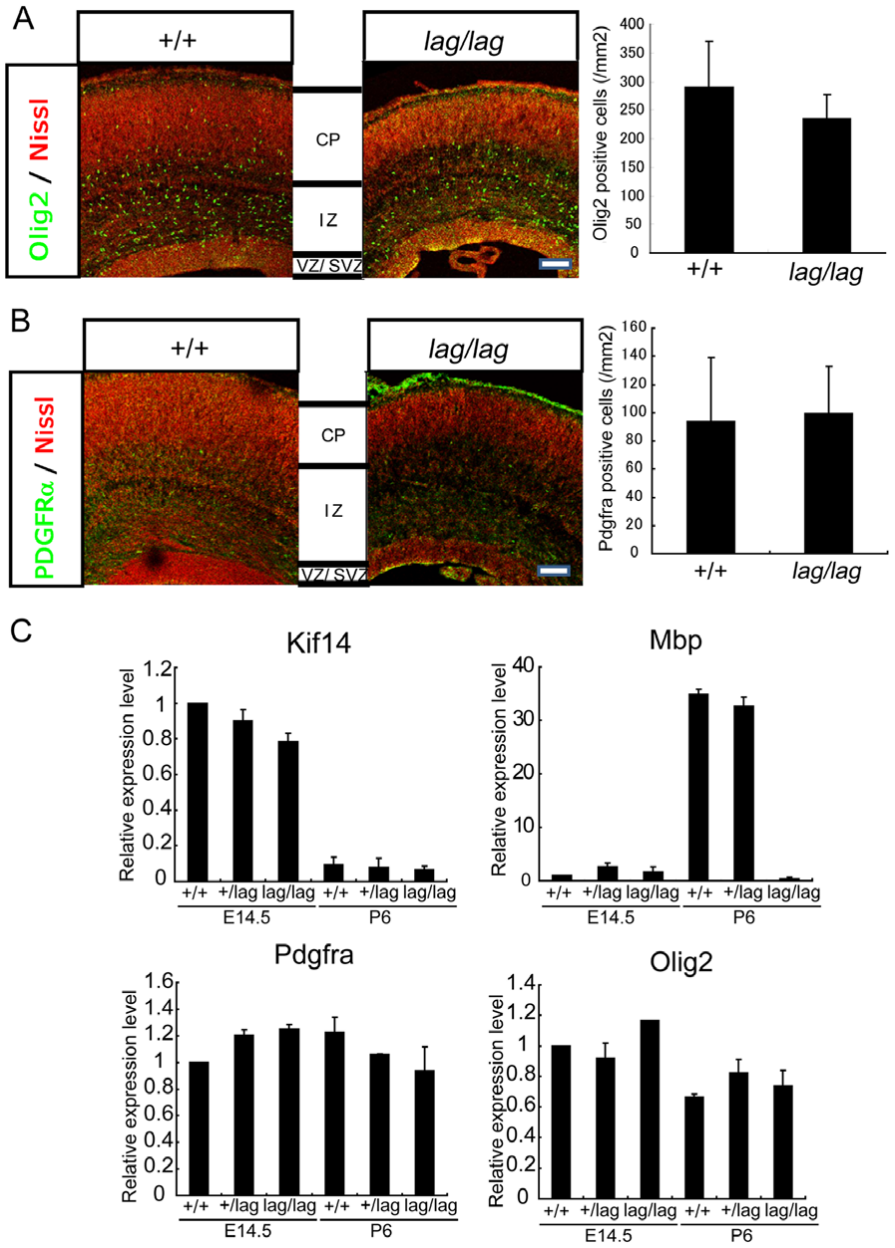
No noticeable difference between OPCs of lag/lag mice and those of wild type mice. (A) Coronal sections of wild-type and *lag/lag* mice cerebral cortex at E17 were counterstained with the fluorescent Nissl stain Neurotrace (red) and immunostained with anti-Olig2 antibody (green). CP, cortical plate; IZ, intermediate zone; VZ/SVZ, ventricular zone/subventricular zone. Bars, 100 µm. The number of Olig2 immuno-positive cells are shown in the right panel (n = 6). Error bars represent SD. (B) Coronal sections of wild-type and *lag/lag* mice cortex at E17 were counterstained with the fluorescent Nissl stain Neurotrace (red) and immunostained with anti-PDGFRα antibody (green). Error bars represent SD. CP, cortical plate; IZ, intermediate zone; VZ/SVZ, ventricular zone/subventricular zone. Bars, 100 µm. The number of PDGFRα immuno-positive cells are shown in the right panel (n = 4). (C) Quantitative real time-PCR analysis of *Kif14*, *Olig2*, *Pdgfra* and *Mbp* in +/+, *lag*/+, *lag*/*lag* mice at E14.5 and P6. Each gene was amplified from whole brain polyA RNA. *β-actin* was used as a positive control for each real time-PCR. Error bars represent SD.

The severe CNS hypomyelination and the dramatically reduced expression of myelin-related genes in *lag* mutants raised the possibility that abnormalities in OPC differentiation might be present in these animals. E17 brain sections were immunostained with anti-Olig2 and anti-platelet derived growth factor receptor-α (PDGFRα) antibodies, markers for OPCs and undifferentiated oligodendrocytes, respectively. We could not detect any significant difference in OPCs or undifferentiated oligodendrocytes in sections of cerebral neocortex of E17 *lag*/*lag* mice compared with littermate controls (Figure 7A, B). Quantitative real-time PCR analysis showed that the levels of *Kif14*, *Pdgfra* and *Olig2* mRNAs were not noticeably different between *lag*/*lag* mice and littermate controls while the expression level of *Mbp* in *lag*/*lag* mice were significantly reduced compared with that in littermate controls (Figure 7C). Collectively, these results suggest that the CNS hypomyelination in *lag* mutants is most likely caused by disruption of oligodendrocyte maturation, rather than a failure to generate OPCs.

### Disrupted Cytoarchitecture of the Cerebellar and Cerebral Cortices and the Hippocampus

**Figure 8:**
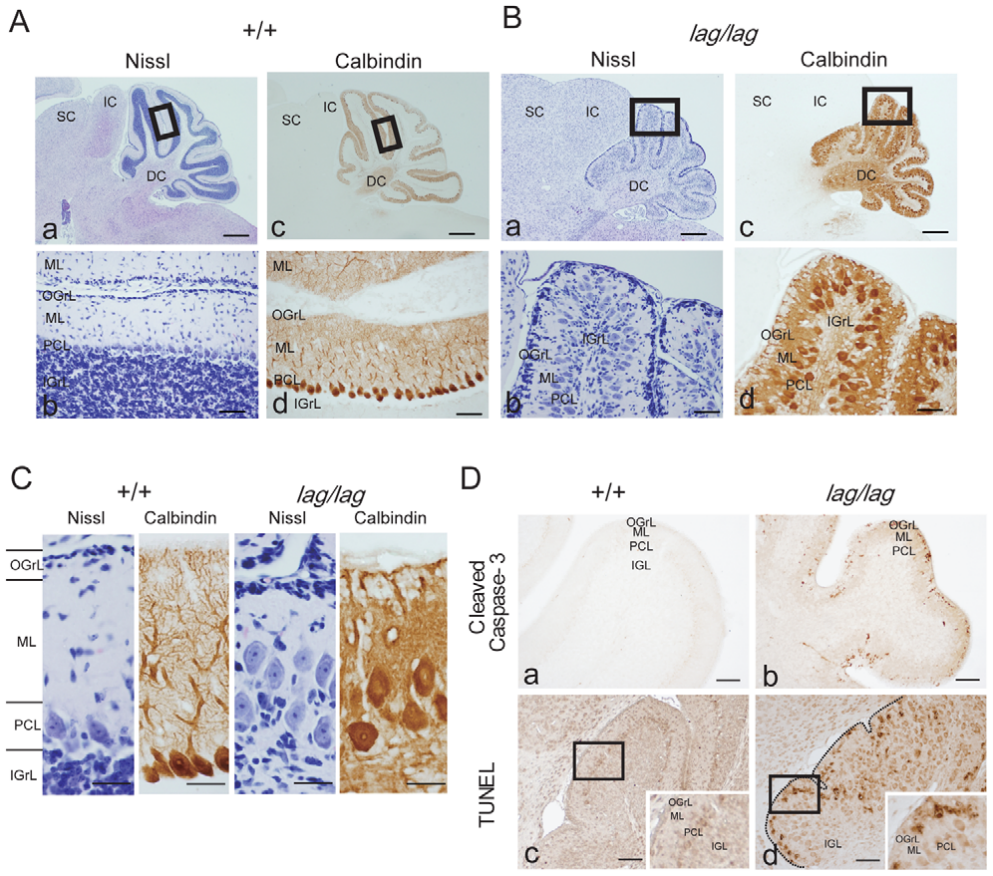
Disrupted cytoarchitecture of the cerebellar cortex of *lag/lag* mice. (A) Littermate wild type cerebellar cortex sagittal sections and the adjoining sections at P14 were counterstained with hematoxylin (Aa and Ab) and immunostained with the anti-calbindin antibody (Ac and Ad). (Ab) and (Ad) are enlarged images of the boxed areas in (Aa) and (Ac), respectively. DC, deep cerebellar nuclei; IC, inferior colliculus; IGrL, internal granule cell layer; ML, molecular layer; OGrL, outer granule cell layer; PCL, Purkinje cell layer; SC, superior colliculus. Bars in (Aa) and (Ac), 1 mm. Bars in (Ab) and (Ad), 100 µm. (B) *lag* mutant cerebellar cortex sagittal sections and the adjoining sections at P14 were counterstained with hematoxylin (Ba and Bb) and immunostained with the anti-calbindin antibody (Bc and Bd). (Bb) and (Bd) are enlarged images of the boxed areas in (Ba) and (Bc), respectively. DC, deep cerebellar nuclei; IC, inferior colliculus; IGrL, internal granule cell layer; ML, molecular layer; OGrL, outer granule cell layer; PCL, Purkinje cell layer; SC, superior colliculus. Bars in (Ba) and (Bc), 1 mm. Bars in (Bb) and (Bd), 100 µm. (C) Laminar structure of the cerebellum. Littermate wild type and *lag* mutant cerebellar cortex sagittal sections and the adjoining sections at P14 were counterstained with hematoxylin and immunostained with the anti-calbindin antibody. Bars, 100 µm. (D) Littermate wild type (Da) and *lag* mutant (Db) cerebellar cortex sagittal sections at P12 were immunostained with the anti-cleaved caspase-3 antibody. Littermate wild type (Dc) and *lag* mutant (Dd) cerebellar cortex sagittal sections at P12 were subjected to TUNEL (terminal deoxynucleotide transferase mediated dUTP nick end labeling) analysis. The insets are enlarged images of the boxed areas. Bars, 100µm.

The size of the *lag*/*lag* cerebellum was much reduced, but cerebellar foliation appeared normal (Figure 8A, B). In *lag*/*lag* mice, Purkinje cell bodies appeared to be scattered, instead of forming an ordered unicellular layer as observed in wild-type mice (Figure 8B, C). In *lag*/*lag* mice, the dendritic arborization of Purkinje cells was not clearly visible by anti-calbindin immunostaining. In wild-type mice, a few granule cells were distributed below the pia mater, whereas many were found in the inner granule cell layer (Figure 8C). In contrast, in *lag*/*lag* mice, many granule cells were arrested in the outer granule cell layer, whereas only a few were localized in the inner granule cell layer. In *lag*/*lag* mice, activated caspase-3 (an apoptotic marker) immunoreactivity was dramatically elevated in the outer granule cell layer compared with wild-type mice (Figure 8D a, b). The cell death occurred in the *laggard* cerebellum was verified using TUNEL staining (Figure 8D c, d).

**Figure 9:**
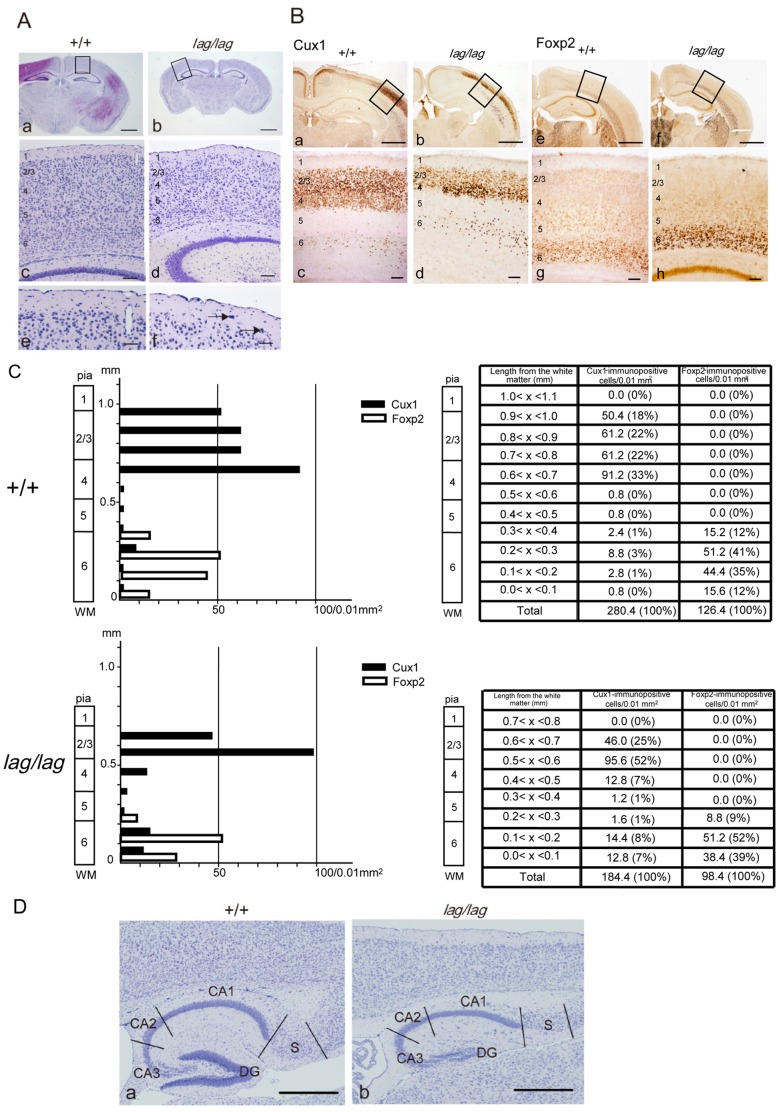
Disrupted cytoarchitecture in the neocortex and hippocampus. (A) Littermate wild type (Aa) and *lag* mutant (Ab) whole brain coronal sections at P14 were counterstained with hematoxylin. (Ac) and (Ad) are enlarged from boxed areas in (Aa) and (Ab), respectively. (Ae) and (Af) are enlarged form (Ac) and (Ad), respectively. The arrows in (Af) indicate ectopic large pyramidal cells in layer 1. Bars in (Aa) and (Ab), 1 mm. Bars in (Ac) and (Ad), 200 µm. Bars in (Ae) and (Af), 100 µm. (B) Littermate wild type (Ba) and *lag* mutant (Bb) whole brain coronal sections at P14 were immunostained with the anti-Cux1. Littermate wild type (Be) and *lag* mutant (Bf) were immunostained with the anti-Foxp2 antibody. (Bc), (Bd), (Bg) and (Bh) are enlarged from boxed areas in (Ba), (Bb), (Be) and (Bf), respectively. Bars in (Ba), (Bb), (Be) and (Bf), 1 mm. Bars in (Bc), (Bd), (Bg) and (Bh), 200 µm. (C) The number of Cux1- and Foxp2-immunopositive cells in the cerebral cortex of the wild type and *lag* mutant mice. Cell counts were performed at 100 µm intervals using a counting grid. (D) Littermate wild type (Da) and *lag* mutant (Db) hippocampal sagittal sections at P14 were counterstained with hematoxylin. CA, Cornu Ammonis; S, subiculum; DG, dentate gyrus. Bars, 1 mm.

To check whether the *kif14* allele could affect cortical development, we measured the thickness of the cerebral cortex of *lag*/*lag* mice. The average thickness of the cerebral cortex, measured on corresponding sagittal and coronal sections, was reduced by 40% in *lag*/*lag* mice (Figure 9A a, b). The laminar structures of the cerebral cortex of *lag*/*lag* mice, especially the supra-granular layers, appeared to be disrupted. The upper margin of layer 2 was not arranged in a laminar fashion in *lag*/*lag* mice (Figure 9A c, d). Ectopic large pyramidal neurons were observed in layer 1 in *lag*/*lag* mice (Figure 9A e, f). To verify whether the layer specificity and the number of cortical neurons could be affect in the mutant, we labeled the cortical neurons with well-known layer 2–4 marker Cux1 and layer 6 marker Foxp2. The number of Cux1 immuno-positive cells in *lag/lag* mice (184.8/0.01 mm^2^) was dramatically reduced compared with those in wild type mice (280.4/0.01 mm^2^) (Figure 9B a–d, C). 5 and 15% of Cux1 immuno-positive cells were observed in the deep cortical layers (layer 6) of wild type and *lag/lag* mice, respectively, suggesting that a part of Cux1 immuno-positive cells remain in the deep cortical layers of *lag/lag* mice. In contrast, the number of Foxp2 immuno-positive cells in *lag/lag* mice (98.4/0.01 mm^2^) was slightly reduced compared with wild type mice (126.4/0.01 mm^2^) (Figure 9B e-h, C). All the Foxp2 immuno-positive cells localized in deep cortical layers both in wild type and *lag/lag* mice. These results suggest that the number and the cell migration of Cux1 positive cells are affected by Kif14 deficiency. In the hippocampus of *lag/lag* mice, the Ammon's horn displayed normal lamination and cell density compared with wild type mice (Figure 9D). In contrast, the cytoarchitecture of the dentate gyrus was disrupted with the granular cell layer not properly formed.

**Figure 10:**
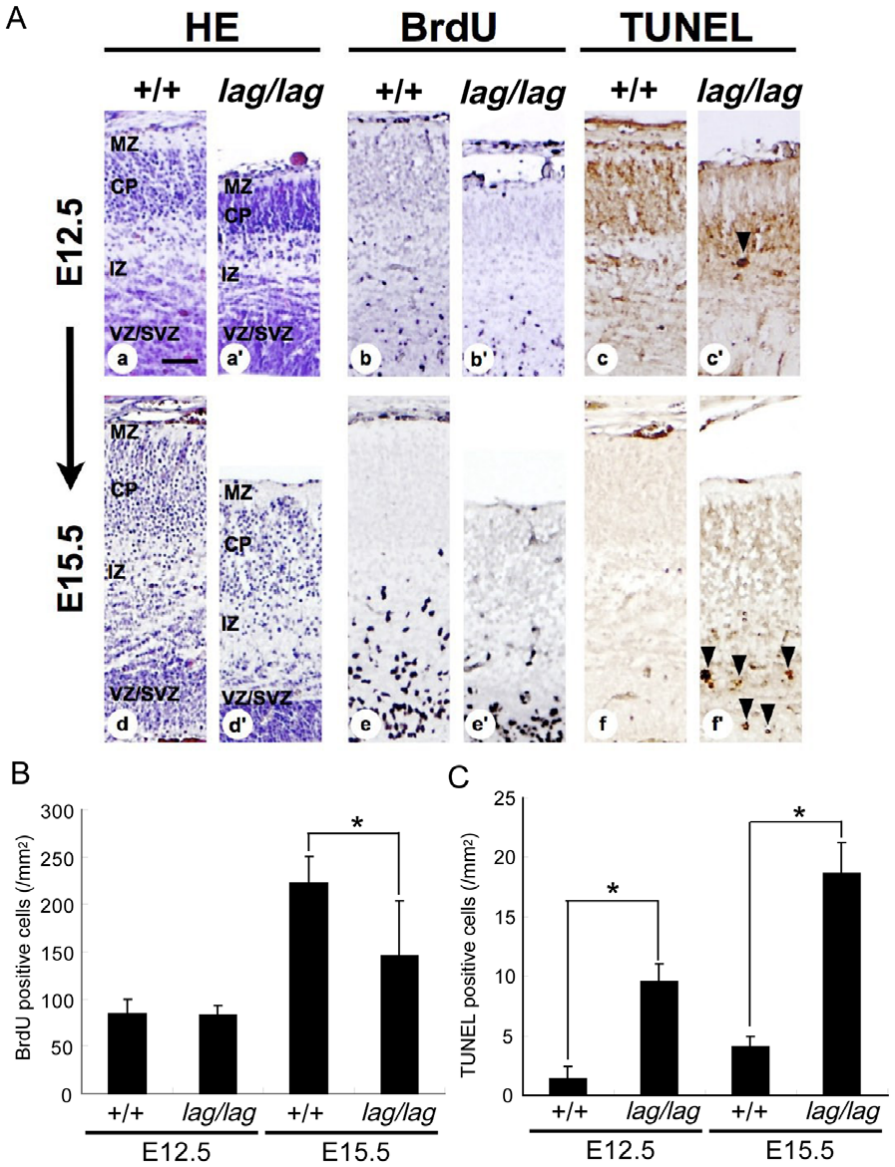
The increased apoptosis and the decreased cell proliferation during development of the cerebral cortex in *lag/lag* mice. (A) Pregnant mice were intraperitoneally injected with BrdU 2 h before sacrifice. Littermate wild type (+/+) (Aa, Ab, Ac, Ad, Ae, Af) and mutant (*lag/lag*) (Aa’, Ab’, Ac’, Ad’, Ae’, Af’) cerebral cortex coronal sections at E12.5 and 15.5 were stained with hematoxylin, anti-BrdU antibody and TUNEL. CP, cortical plate; IZ, intermediate zone; MZ, marginal zone; VZ/SVZ, ventricular zone/subventricular zone. Bars, 100 µm. (B) The number of BrdU immuno-positive cells (n = 4). Error bars represent SD. Asterisk indicates statistical significance (*t* test; *, p<0.05). (C) The number of TUNEL immuno-positive cells in (n = 4). Cell counts were performed in the sensory-motor cortex. Error bars represent SD. Asterisks indicate statistical significance (*t* test; *, p<0.01).

The reduced thickness of the cerebral cortex could be caused by a decreased rate of cell proliferation, apoptosis or a combination of these two processes. To determine the cause of the *lag*/*lag* brain phenotype, we conducted BrdU and TUNEL experiments at E12.5, and E15.5, and quantified the number of BrdU and TUNEL-positive cells. To do this, pregnant mice were injected with BrdU intraperitneally 2 hr before sacrifice. A series of serial coronal sections of embryonic cortices were immunostained with anti-BrdU antibody. In E12.5 mice cortex, the cell proliferation rate is not noticeable different between mutant mice and wild type mice while the apoptotic cell death in mutant mice increased in number compared with wild type mice (Figure 10). On the other hand, in E15.5 mice cortex, the cell proliferation rate in mutant mice reduced and the apoptotic cell death increased compared with wild type mice. The number of apoptotic cells in E15.5 mice was almost two times higher than that in E12.5 mice. The decreased rate of cell proliferation in E15.5 mice could be caused by an apoptosis at E12.5 mice. This increased apoptotic cell death is thought to be responsive for the decreased number of Cux1 and Foxp2 immuno-positive cells in mutant mice. Especially, reduction of Cux1 immuno-positive cells of the supragranular cortical neurons could be due to the increased apoptotic cell death in E15.5 mice. In contrast, apoptotic cells were not detected in *lag*/*lag* mice at P14 using the TUNEL method (Figure S5). Together, these results indicate that the disrupted cytoarchitecture of the cerebellar and cerebral cortices results from apoptotic cell death at neonatal stage. Thus, the decreased brain size in *lag*/*lag* mice is likely caused by a dramatic increase in cell death. Alternative possibility is that the increased apoptosis and the decreased rate of cell proliferation could occur simultaneously in *lag*/*lag* mice, resulting in the abnormally small head.

## Discussion

In this study, we describe a novel neurological mouse mutant *laggard* (*lag*) exhibiting reduced brain size, ataxia and premature death. While the entire brain and the spinal cord are reduced in size, certain areas such as the cerebral cortex and cerebellum are disproportionately smaller. Although a decrease in *lag/lag* body weight was also measurable after the first week, this is likely to be a consequence of poor feeding rather than a direct effect of the mutation. We discovered that there is a dramatic increase in cell death within the brains of *lag/lag* mice at the same time at which the reduction in brain size becomes evident.

Mice homozygous for the *lag* mutation exhibit a severe CNS hypomyelination, This hypomyelination in *lag* mutants is most likely caused by disruption of oligodendrocyte maturation, but the underlying mechanisms of hypomyelination remain unknown. A biogenesis of the myelin sheath requires transport of large amounts of myelin components to achieve the spiral wrapping of the resulting membrane around the axon [23]. The kinesin motor family proteins play a role in controlling these processes. For example, Kif1b is required to deliver myelin mRNA to the myelinating processes of oligodendrocytes, to elaborate the correct amount of myelin around axons [24]. Other kinesin motor family proteins are supposed to transport various components such as vesicle, mitochondria and protein complexes along the microtubule to control elongation and polarization of myelinating processes [25]. Kif14 contains long C-terminal tail domain. The function of C-terminal tail domain remains unknown. Some cargos essential for myelination might bind to the C-terminal tail domain directly or indirectly and regulate the myelin sheath formation.

The PI3K/AKT/mTOR signaling pathway for cell survival plays an important role in myelination by oligodendrocytes [26]. Transgenic mice with forced expression of activated Akt in oligodendrocytes develop dramatic hypermyelination and mTOR activation has also been reported to promote oligodendrocyte maturation [27]. Mice deficient for Pten, negative regulator of Akt, exhibit dramatic hypermyelination in the spinal cord and corpus callosum [28]. In this study, Kif14 deficient mice show hypomelynation and apoptosis. These previous reports and our results raise the possibility that Kif14 controls myelination by oligodendrocytes and neuronal cell survival by regulating PI3K/AKT/mTOR signaling pathway directly or indirectly.

In this study we showed that Kif14 mutation causes the disrupted cytoarchitecture of dentate gyrus. One possible explanation is that apoptosis reduced the number of hippocampal granule precursor cells in the ventricular zone (VZ) during fetal stage (E12.5–15.5). The reduction in hippocampal granule precursor cells, which migrate from the VZ to the subgranular zone (SGZ) of the dentate gyrus [29], may lead to dentate gyrus hypoplasia.

On the other hand, migrated granule cells rapidly proliferate in the SGZ at first postnatal week, resulting in the dramatically thickness of the dentate gyrus [30]. Kif14 has been reported to be involved in efficient cytokinesis in cooperation with Citron kinase [20]. In addition, it is also reported that expression of Kif14 is up-regulated in the cerebeller granule cell-derived medulloblastoma [31]. Kif14 may be necessary for rapid cytokinesis and cell proliferation in granule cells while cytokinesis of pyramidal cells is less affected by Kif14 deficiency. Although we did not analyze neonatal mice in this study, Kif14 deficiency may cause cytokinesis failure of granule cells. Subsequently, aberrant cell division may induce apoptosis. Indeed, in the Citron kinase knockout mice, the disrupted cytoarchitecture of dentate gyrus is observed [32]. A significant number of tetraploid neurons are observed and these abnormal neurons are supposed to induce apoptotic cell death.

The high expression levels of Citron kinase were observed in the neural tube, and the internal and external germinal layers of the cerebellum [32]. In contrast low expression was detected in the other somatic organs. Our western blotting analysis showed that Kif14 was highly expressed during E12.5–16.5, and its expression stage is similar to that of Citron kinase [32]. This raises the possibility that similar expression patterns might be observed between Kif14 and Citron kinase during early developmental stage. At present our anti-Kif14 antibody specificity is rather poor, resulting in the difficulty in immunostaining of Kif14 in the fetal brain. In future, we are planning to develop Kif14 specific antibody to detect the intercellular localization of Kif14 and uncover the underlying mechanism of hypomyelination caused by Kif14 deficiency.

In summary, we describe a novel neurological mutant, *lag*, that displays extensive and brain-specific cellular degeneration. Degeneration has also been observed in a number of other neurological mutants. Although similar to other mutants in this regard, the pattern of neuronal death in *lag/lag* mice is unique. This mutation affects diverse populations of cells that appear to share no functional relationship, and cell loss occurs throughout the brain. Several developmental events, such as proliferation, differentiation and migration, are underway during the period in which cell death is observed in *lag/lag* mice. It is likely that the mutation affects one of these important processes. Regardless of the actual process that is affected, the unique phenotype displayed by *lag* renders it a valuable tool for investigating the mechanisms that regulate later stages of normal development of the nervous system and for identifying the molecular mechanisms involved. Because virtually every region of the brain is affected, the mutation likely affects a common and fundamental process. As reduced brain size is also seen in humans with microcephaly [33], [34], and aberrant neuronal loss due to apoptosis is seen in a number of neurodegenerative diseases, the *lag* mutation could shed valuable insight into the pathogenetic mechanisms underlying various neuropathologies.

## Supporting Information

Figure S1 Reduced body and brain weight of *lag* mouse (A) Body weight. A graph of body weights of the littermate normal control mice (black bars) and the *lag* mutant mice (white bars) from P1 to P13 (normal control P1, n = 12; P5, n = 11; P9, n = 16; P13, n = 9; *lag* P1, n = 4; P5, n = 5; P9, n = 6; P13, n = 3). Error bars represent SD. (B) Brain weight. A graph of brain weights of the littermate normal control mice (black bars) and the *lag* mutant mice (white bars) from P1 to P13 (normal control P1, n = 12; P5, n = 11; P9, n = 16; P13, n = 9; *lag* P1, n = 4; P5, n = 5; P9, n = 6; P13, n = 3). Error bars represent SD.
(TIF)

Figure S2 Expression and cellular localization of the three *Kif14* transcripts identified in the *lag*/*lag* mouse. (A) Schematic structures of Kif14 and three truncated forms in the *lag*/*lag* mouse. Red, yellow and green regions correspond to motor domains, Fork head association (FHA) domain and coiled coil, respectively. Full-length and three aberrant truncated *kif14* (Δexon5, Δexon5–6 and Δexon5∶11 bp) cDNAs, were subcloned into the pCMV-FLAG vectors. (B) Western blot analysis of various Flag-Kif14 proteins expressed in HEK293 cells. Cell extracts were subjected to SDS-PAGE, followed by immunoblotting with the anti-Kif14 rabbit polyclonal antibody. (C) Various Flag-Kif14 constructs were expressed in NIH3T3 cells. Cells were immunostained with anti-Flag (green) and anti-α tubulin (red) antibodies. Bar, 10 µm.
(TIF)

Figure S3 Western blot analysis of Kif14 protein in *lag/lag* mice using higher percentage acrylamide gels. Extracts (20 µg of proteins) were prepared from E13.5 mouse whole brain (+/+, *lag*/+, *lag*/*lag*). The samples were subjected to SDS-PAGE (8% gel), followed by immunoblotting with the anti-Kif14 rabbit polyclonal antibody. We could not detect truncated forms of Kif14 from the three *Kif14* transcripts identified in the *lag/lag* mouse. The arrow indicates full-length Kif14. The asterisks indicate the non-specific bands.
(TIF)

Figure S4 Western blot analysis of Kif14 from different developmental stages of wild-type mouse. Protein extracts (30 µg of proteins) were prepared from mouse whole brains at E12.5, E14.5, E16.5, P1, and P13. The samples were subjected to SDS–PAGE (5% gel) and then transferred to a nitrocellulose membrane. The membrane was immunoblotted with the anti-Kif14 rabbit polyclonal antibody. The arrow indicates Kif14 bands. The asterisk indicates the non-specific bands.
(TIF)

Figure S5 TUNEL (terminal deoxynucleotide transferase mediated dUTP nick end labeling) analysis of the neocortex and hippocampus. (A) Littermate wild type (Aa) and *lag* mutant (Ab) coronal sections of the neocortex at P14 were subjected to TUNEL analysis. (B) Littermate wild type (Ba) and *lag* mutant (Bb) hippocampal sagittal sections at P14 were subjected to TUNEL analysis. CA, Cornu Ammonis; DG, dentate gyrus. Bars, 200 µm.
(TIF)

Video S1 14 days old *laggard* mouse. Mutant mouse displays tremor and ataxia.
(MPEG)
