# Supplementary figures and images for: Kif14 Mutation Causes Severe Brain Malformation and Hypomyelination
Source: PLoS One. 2013 Jan 4;8(1):e53490. doi: 10.1371/journal.pone.0053490 (PMC3537622; doi:10.1371/journal.pone.0053490)

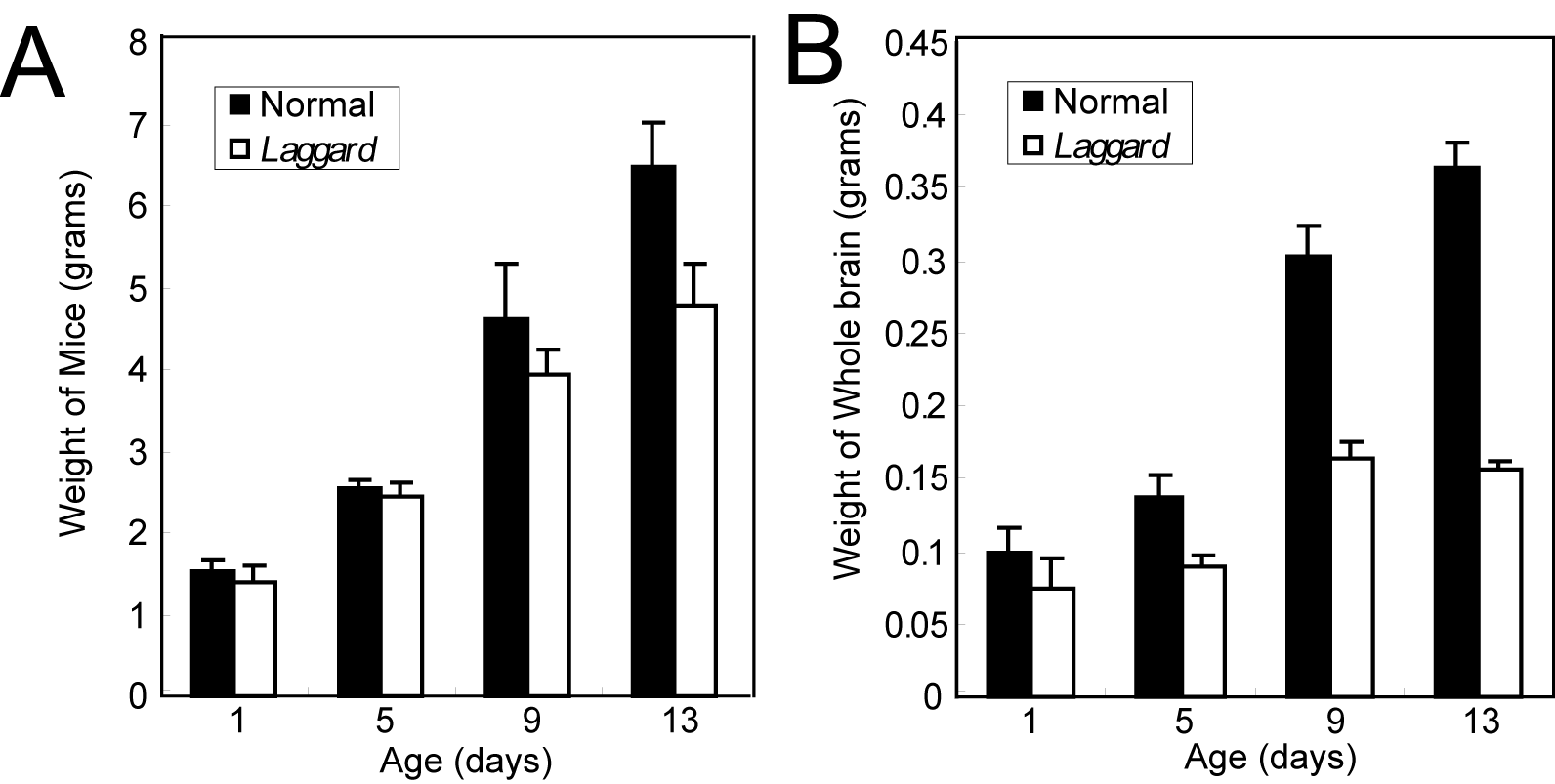

Supplement: Figure S1 — Reduced body and brain weight of lag mouse (A) Body weight. A graph of body weights of the littermate normal control mice (black bars) and the lag mutant mice (white bars) from P1 to P13 (normal control P1, n = 12; P5, n = 11; P9, n = 16; P13, n = 9; lag P1, n = 4; P5, n = 5; P9, n = 6; P13, n = 3). Error bars represent SD. (B) Brain weight. A graph of brain weights of the littermate normal control mice (black bars) and the lag mutant mice (white bars) from P1 to P13 (normal control P1, n = 12; P5, n = 11; P9, n = 16; P13, n = 9; lag P1, n = 4; P5, n = 5; P9, n = 6; P13, n = 3). Error bars represent SD. (TIF) [file pone.0053490.s001.tif]

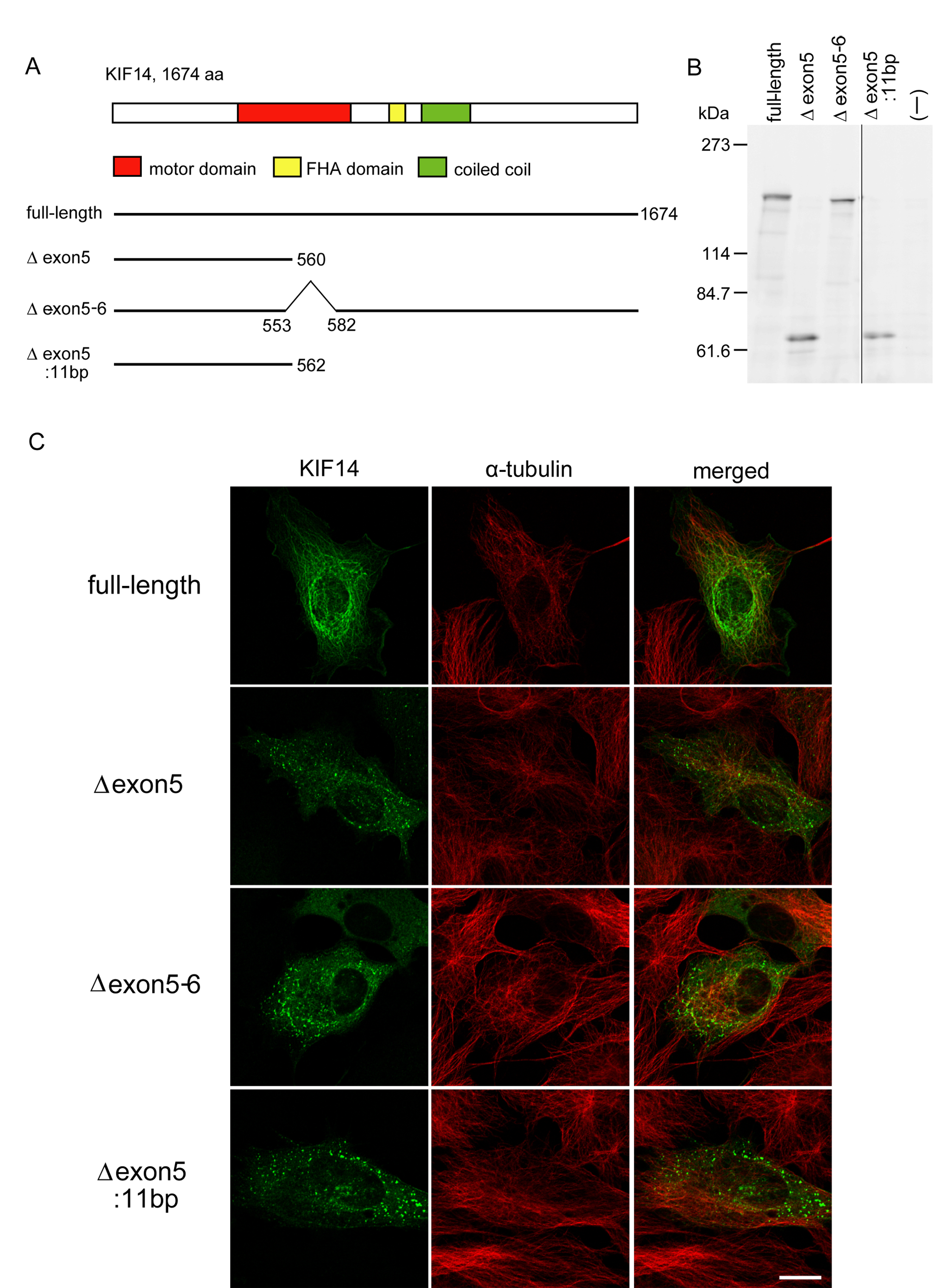

Supplement: Figure S2 — Expression and cellular localization of the three Kif14 transcripts identified in the lag/lag mouse. (A) Schematic structures of Kif14 and three truncated forms in the lag/lag mouse. Red, yellow and green regions correspond to motor domains, Fork head association (FHA) domain and coiled coil, respectively. Full-length and three aberrant truncated kif14 (Δexon5, Δexon5–6 and Δexon5∶11 bp) cDNAs, were subcloned into the pCMV-FLAG vectors. (B) Western blot analysis of various Flag-Kif14 proteins expressed in HEK293 cells. Cell extracts were subjected to SDS-PAGE, followed by immunoblotting with the anti-Kif14 rabbit polyclonal antibody. (C) Various Flag-Kif14 constructs were expressed in NIH3T3 cells. Cells were immunostained with anti-Flag (green) and anti-α tubulin (red) antibodies. Bar, 10 µm. (TIF) [file pone.0053490.s002.tif]

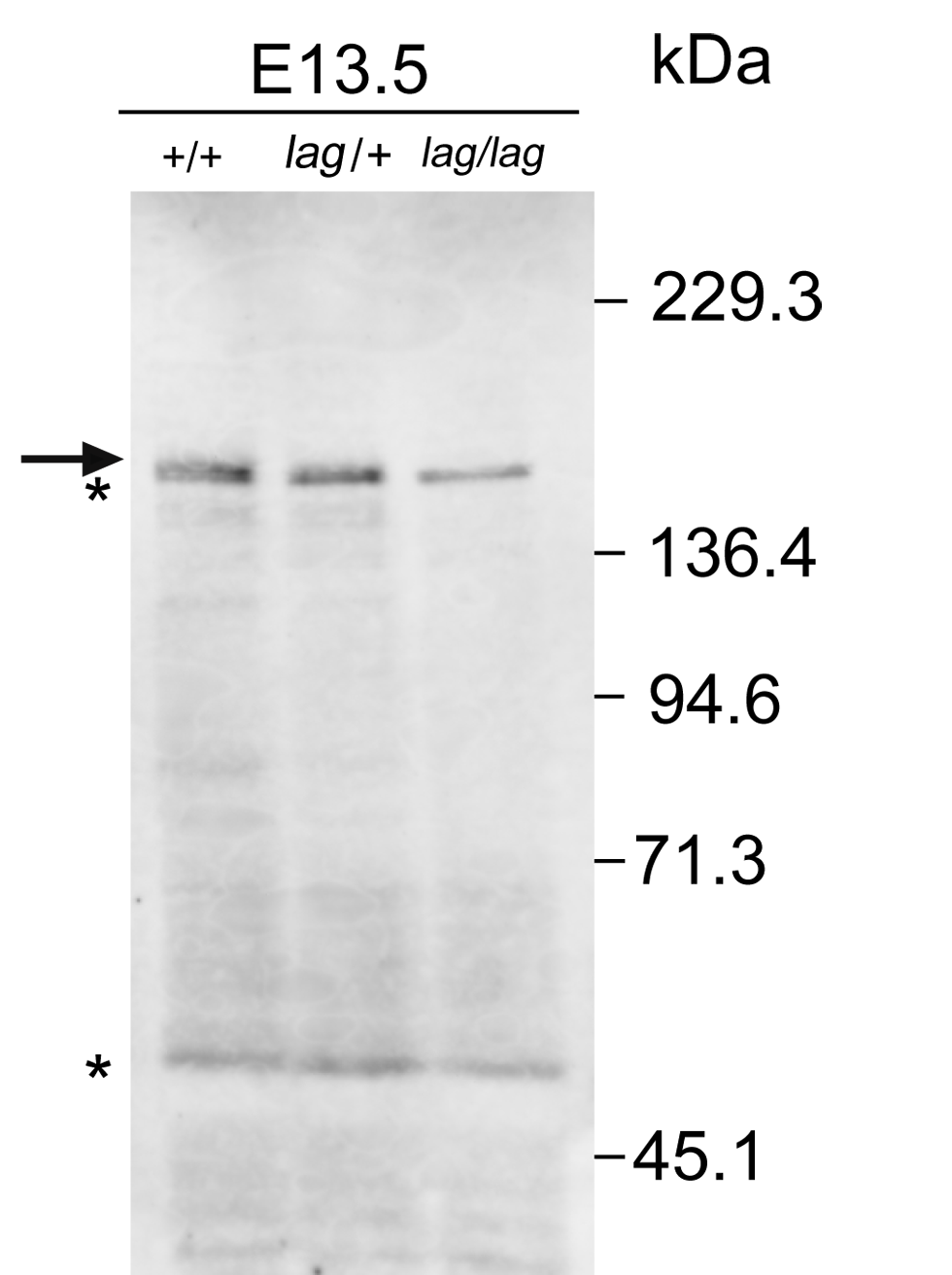

Supplement: Figure S3 — Western blot analysis of Kif14 protein in lag/lag mice using higher percentage acrylamide gels. Extracts (20 µg of proteins) were prepared from E13.5 mouse whole brain (+/+, lag/+, lag/lag). The samples were subjected to SDS-PAGE (8% gel), followed by immunoblotting with the anti-Kif14 rabbit polyclonal antibody. We could not detect truncated forms of Kif14 from the three Kif14 transcripts identified in the lag/lag mouse. The arrow indicates full-length Kif14. The asterisks indicate the non-specific bands. (TIF) [file pone.0053490.s003.tif]

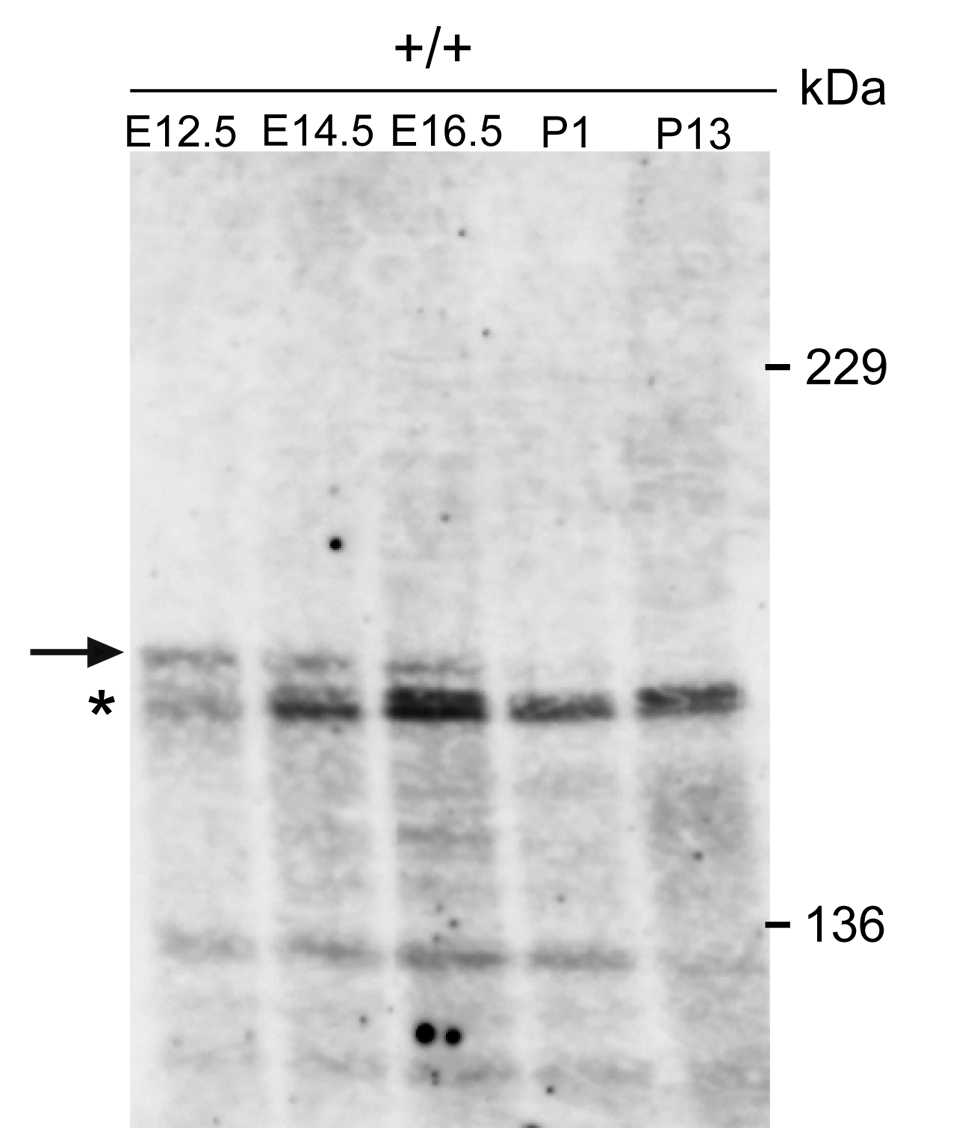

Supplement: Figure S4 — Western blot analysis of Kif14 from different developmental stages of wild-type mouse. Protein extracts (30 µg of proteins) were prepared from mouse whole brains at E12.5, E14.5, E16.5, P1, and P13. The samples were subjected to SDS–PAGE (5% gel) and then transferred to a nitrocellulose membrane. The membrane was immunoblotted with the anti-Kif14 rabbit polyclonal antibody. The arrow indicates Kif14 bands. The asterisk indicates the non-specific bands. (TIF) [file pone.0053490.s004.tif]

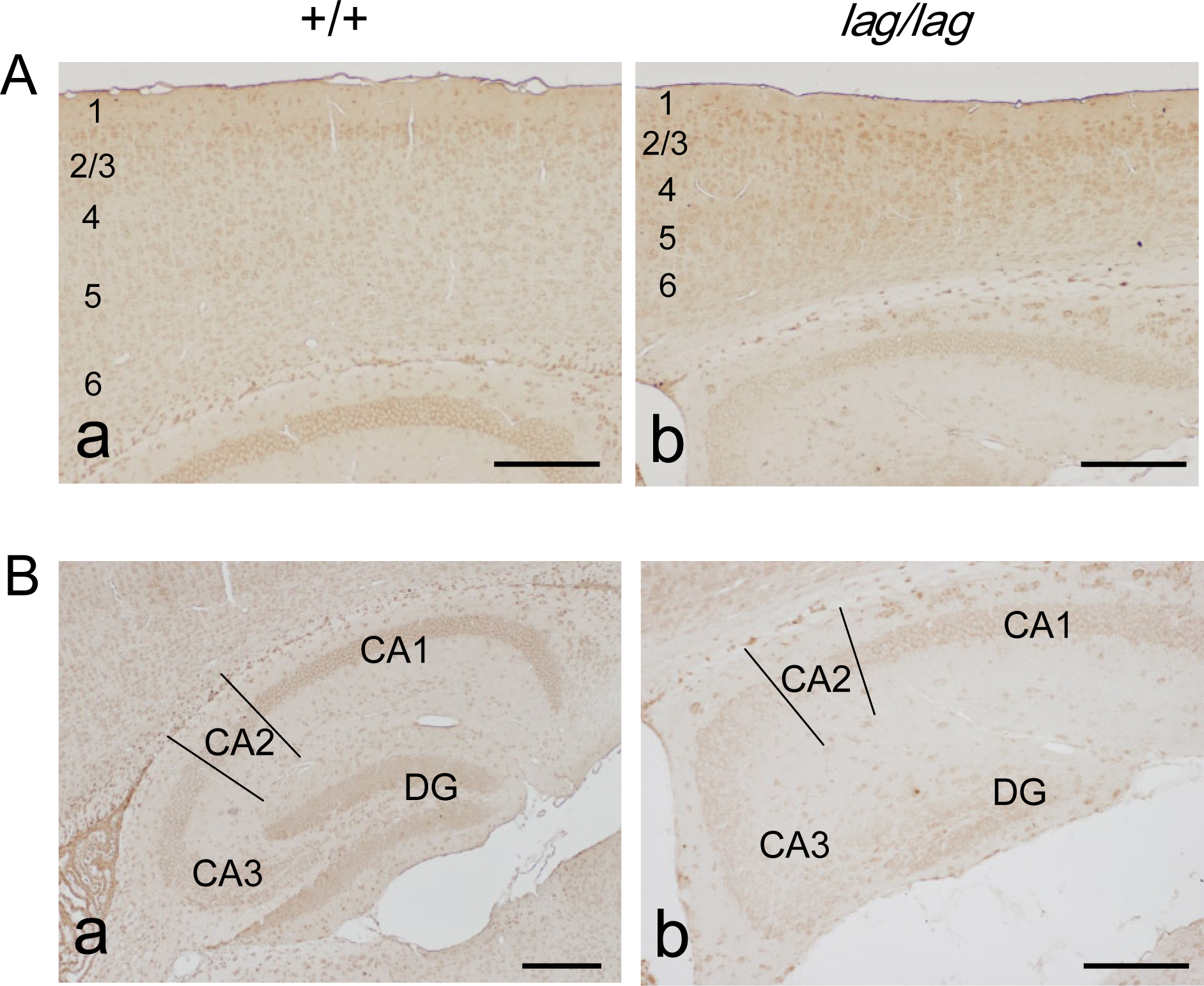

Supplement: Figure S5 — TUNEL (terminal deoxynucleotide transferase mediated dUTP nick end labeling) analysis of the neocortex and hippocampus. (A) Littermate wild type (Aa) and lag mutant (Ab) coronal sections of the neocortex at P14 were subjected to TUNEL analysis. (B) Littermate wild type (Ba) and lag mutant (Bb) hippocampal sagittal sections at P14 were subjected to TUNEL analysis. CA, Cornu Ammonis; DG, dentate gyrus. Bars, 200 µm. (TIF) [file pone.0053490.s005.tif]
